# Engineered Polymeric Materials for Biological Applications: Overcoming Challenges of the Bio–Nano Interface

**DOI:** 10.3390/polym11091441

**Published:** 2019-09-02

**Authors:** Joshua D Simpson, Samuel A Smith, Kristofer J. Thurecht, Georgina Such

**Affiliations:** 1 Centre for Advanced Imaging, Australian Institute for Bioengineering and Nanotechnology, ARC Centre of Excellence in Convergent Bio-Nano Science and Technology and ARC Training Centre for Innovation in Biomedical Imaging Technology, the University of Queensland, St Lucia QLD 4072, Australia; j.simpson@uq.edu.au; 2 School of Chemistry, University of Melbourne, Parkville VIC 3010, Australia; smiths2@student.unimelb.edu.au

**Keywords:** nanomedicine, biodistribution, nanoparticle fate, cellular compartmentalization, cellular trafficking

## Abstract

Nanomedicine has generated significant interest as an alternative to conventional cancer therapy due to the ability for nanoparticles to tune cargo release. However, while nanoparticle technology has promised significant benefit, there are still limited examples of nanoparticles in clinical practice. The low translational success of nanoparticle research is due to the series of biological roadblocks that nanoparticles must migrate to be effective, including blood and plasma interactions, clearance, extravasation, and tumor penetration, through to cellular targeting, internalization, and endosomal escape. It is important to consider these roadblocks holistically in order to design more effective delivery systems. This perspective will discuss how nanoparticles can be designed to migrate each of these biological challenges and thus improve nanoparticle delivery systems in the future. In this review, we have limited the literature discussed to studies investigating the impact of polymer nanoparticle structure or composition on therapeutic delivery and associated advancements. The focus of this review is to highlight the impact of nanoparticle characteristics on the interaction with different biological barriers. More specific studies/reviews have been referenced where possible.

## 1. Introduction

Nanomedicine has emerged as an important strategy for improving cancer treatment due to the ability of nanoparticles to encapsulate therapeutic cargo and deliver it more specifically and more effectively to a treatment site. Significant research in this area has demonstrated that nanoparticle formulations provide important benefits over systemically-delivered drugs alone, including improving efficacy and safety profiles. Nanoparticle formulations also offer the potential to increase the range of therapeutics to biologic drugs, such as oligonucleotides or proteins, or combine multiple therapeutic cargo within the one carrier. These properties have been demonstrated in a number of successful nanomedicines either in clinical trials or have been approved by the U.S. Food and Drug Administration (FDA). A well-known example is Abraxane®, which was approved by the FDA in 2005 [1]. This nanoparticle involves the conjugation of paclitaxel with albumin, demonstrating improved pharmacokinetics, enhanced tumor inhibition, and reduced side effects when treating a variety of refractory malignancies compared to the drug alone. The development of new nanomedicines continue to increase and spans a wide range of different types of carriers from drug conjugates with stealth polymers such as poly(ethylene glycol) (PEG) through to inorganic, protein, or polymer nanoparticles. Material scientists have developed a number of tools to tailor the interactions of the nanocarriers with their biological surroundings, including tuning morphological characteristics, such as size and shape, as well as modulating the chemical signature of such materials, in particular the surface chemistry. There is evidence to suggest nanoparticles can reach the target site (e.g., a specific tumor) using the enhanced permeability and retention (EPR) effect due to the leakiness of vasculature in these regions. However, this can be specific to certain tumor types and thus targeting specific proteins on tumors is also an important research area. Nanoparticles can also be designed to recognize specific cancers and to release drugs via specific cellular triggers, such as variations in pH or particular enzyme profiles. The characteristics of the nanoparticle delivery system needs to be tailored for specific applications and delivery methods. In this perspective we will highlight the challenges associated with designing nanocarriers for cancer therapy using intravenous (IV) administration.

**Figure 1:**
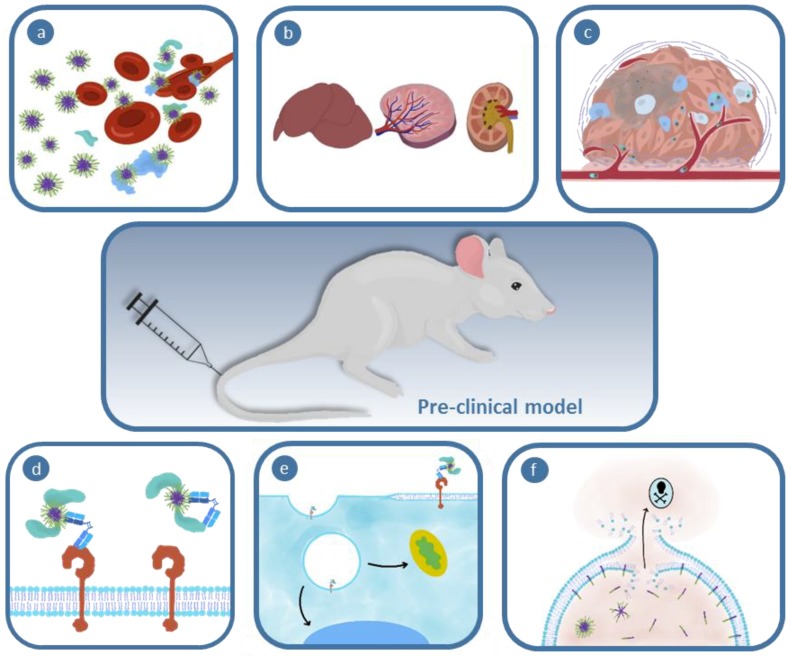
Schematic overview of key biological barriers encountered by polymeric nanomedicines. (**a**) Interactions that occur immediately post-injection with components of blood and plasma; (**b**) biodistribution of polymers and clearance mechanisms; (**c**) barriers that occur at the tumor site and once a material has gained access to the tumor volume; (**d**) receptor accessibility and ability of associated ligands to bind and activate the target surface protein; (**e**) internalization and subsequent intracellular trafficking behaviors; and (**f**) requirement for therapeutic escape from vesicular compartments.

While many nanoparticle formulations show promise in vitro (cell culture), or even using preclinical mouse models in vivo, the number of nanomedicines translated into clinical use remains low [2]. One rationale for the low rates of clinical translation is there is still limited understanding of how nanoparticle structure governs the interaction with the different biological environments and thus their ability to migrate biological roadblocks in order to successfully deliver a drug. These roadblocks begin as soon as the particles are injected, as they must evade interactions with other biologics within the blood and subsequent opsonization. They must then be capable of extravasation from the blood stream into the tissue and penetration into the tumor environment. A number of studies have demonstrated these processes have limited efficacy. Recently, Chan et al. [3] evaluated nanoparticle delivery to tumors in a variety of murine models through meta-analysis of a broad body of literature, showing the median delivery to the tumor was invariably low (0.7% of injected dose). This highlights the significant challenges associated with navigating the many barriers that prevent tumor accumulation following intravenous injection of nanomedicines. But the effectiveness of nanomedicines is not limited to tumor tissue deposition; the tumor microenvironment is heterogeneous and complex, therefore targeting a specific cell population within this environment offers an idealistic strategy for improving therapeutic efficacy. This may then facilitate other stringent requirements often necessary in drug therapies, including nanoparticle internalization into cells, disassembly to release its cargo, and then delivery of the therapeutic to the site of action for a particular drug. This is a particularly challenging part of the process, especially for biological drugs that have generated interest in recent years. This is because nanoparticles are typically delivered into the endosomal/lysosomal compartment, which is not the site of action for most drugs. Therefore, nanoparticles must undergo “endosomal escape”. Recent literature has shown even effective nanoparticles facilitate only small amounts of endosomal escape (1–2%) [4]. Therefore, while we have many clinical systems that show efficacy, it is clear that we could improve the payload going to the correct region of a tumor in order to create better therapies. In order to design more effective delivery systems, an important first step is to understand how nanoparticle structure can be tuned to optimize their ability to migrate each biological challenge. While there is a broad range of nanomedicines in the literature that show promise for clinical application, in this article we will focus on polymeric nanoparticles in the order of 1 to 200 nm. Important reviews on inorganic nanoparticles or polymer conjugates can be found in the following references [5,6]. Polymeric nanomedicines are attractive as they offer versatile syntheses, where the size, shape, and composition of the particles can be tuned to offer control over biological interactions. For example, it has been demonstrated that nanoparticle size and shape impacts clearance rate. In this perspective, we will discuss the impact of biophysical properties of polymer nanoparticles as they traverse the various biological barriers to enable efficient drug delivery. We focus on blood and plasma interactions, clearance, extravasation, and tumor penetration, through to cellular targeting, internalization, and endosomal escape, as shown schematically in Figure 1. The literature in each of these areas (summarized in Table 1) shows that understanding the impact of nanoparticle structure on biological interactions provides insight into how to better design polymeric nanoparticles for nanomedicine; the impact of this understanding will only be enhanced as we learn more about these structure-roperty relationships.

## 2. Interactions in the Blood Stream

One of the key factors that drives enhanced accumulation of polymeric nanoparticles in tumor tissue is their prolonged circulation time in blood. While it is well-recognized that longer circulating particles provide greater opportunity for the therapeutic cargo to extravasate into tumor tissue, it also has the effect of prolonging contact and exposure to blood components leading to immunological effects [7]. In addition to the activation of platelets, intravenously-injected polymeric nanocarriers can induce coagulation cascade events owing to activation of specific enzymes. Perhaps the most detrimental event for nanocarriers is activation of the complement cascade, initializing rapid clearance of “foreign” bodies by the mononuclear phagocytic system (MPS) following opsonization [8]. Evidence suggests polymeric nanoparticles do not cause an antigen-specific T cell response (at least for the most common polymeric systems utilized in nanomedicine). However, their presence likely has an indirect effect on adaptive immune responses, most likely through production of inflammatory cyto- and chemokines and promotion of dendritic cell maturation [9].

**Figure 2:**
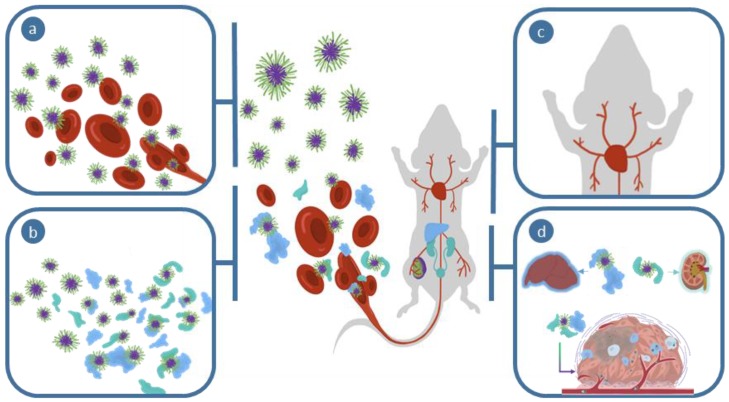
Schematic depicting the interaction between opsonization, biodistribution, and clearance pathways of injected polymer nanomedicines. (**a**) Polymeric materials enter the bloodstream (**b**) become coated with opsonins and other biomolecules during (**c**) systemic transport; a combination of polymer properties and biological interactions lead to (**d**) clearance from the system or accumulation within organs or tumor tissue.

Within seconds of entering the blood stream, nanomaterials are bombarded with an array of compounds endogenous to plasma [10] (Figure 2). Many of these plasma constituents then non-covalently bind to the surface of the polymers to form the protein corona [11]. The formation and dynamic exchange within this coating of adsorbed biological molecules remains ambiguous; however, is believed to possess a specific two-layer morphology [12]. The resulting biomolecular-corona is commonly divided into two layers, the hard and soft corona, referring to the inner and outer proteinaceous shells, respectively [13,14]. The binding proteins and biomacromolecules, broadly denoted as opsonins, modulate the behavior of the potential nanomedicine in vivo, as they are able to signal to the immune system [15], dictate clearance pathways [12], and alter downstream events required for efficacious therapeutic delivery [16]. This initial response to nanomedicine administration is a major contributor for dictating effective distribution, cellular interaction, and ultimate efficacy of nanoparticle delivery systems.

### 2.1. Opsonization

#### 2.1.1. The Protein Corona

Opsonization remains one of the most commonly discussed biological obstacles known to impact the translation of nanoparticles from animal models to the clinical setting [17]. The dynamic process of binding and exchange of plasma proteins associated with the nanomaterial surface has been demonstrated to play a key role in the clinical translation of materials. It has been shown that some protein modifications produce improved cellular uptake at the tumor site [18], whereas others induce immune responses leading to clearance via the mononuclear phagocytic system (MPS) [15]. Several opsonin components have been identified, including complement proteins [15], apolipoproteins [10], and various metabolites [19], with the adsorption of specific constituents or ratios being responsible for stealth effects (or lack thereof) in PEG-based materials [10]. The combination of nanoparticle-protein [20] and protein-protein [21] interactions leads to a vast array of different protein corona composition. Numerous physicochemical factors have been shown to contribute to the accumulation and subsequent composition of the protein corona including chemical composition [22], size [23], curvature [22], rigidity [24], hydrophobicity [22], presence of protein targeting ligands [25], and surface characteristics [10,11,26]. These factors contribute to the unpredictable outcome of interactions between nanoparticles and physiological phenomena, with enhanced variability also arising due to flow rate encountered in different parts of the animal [27], enzymatic modifications [12], glycosylation state [28], as well as localized plasma protein concentration [27] and content [12] within anatomical regions. While the interaction with blood proteins has been profiled for certain kinds of materials (e.g., gold nanoparticles [29] and liposomes [30]), proteomic fingerprinting has not been done for polymeric nanomedicines (at least under conditions that match those present within the blood stream of an animal). This is due to difficulty in extracting intact complexes and the fragility of the soft corona [14]. A further confounding issue with studying the recruitment and influence of opsonins on materials is the enormous degree of inter-patient variability in the protein content of the plasma [31]. The diverse and flexible nature of the plasma proteome is among the primary difficulties in predicting the behavior of materials in clinical trials [31]. Even minor details of the patient’s life history can produce a suite of changes in their plasma proteome. It is highly likely that wide-ranging variations in the opsonins present on polymer nanoparticles exist. With circulating plasma proteins known to vary between individuals [32,33,34,35], translational modifications such as glycosylation [36] can provide even greater diversity in participation of circulating proteins and alter their binding properties and affinities.

#### 2.1.2. Measuring and Monitoring Opsonization

Due to the impact of opsonization on nanomaterials, a number of tools have been developed to better characterize participating components of plasma and predict the outcome of the interaction. The techniques and tools for characterizing this biological barrier have been reviewed elsewhere [37,38]. With new approaches rapidly being developed and our knowledge of these systems improving, new strategies for avoiding specific components or controlling this process are regularly being reported. Many nanomedicine adaptations have been generated through pre-incubation with serum [39] and artificial creation of the hard corona [40], whereas others have used polymer design to modulate protein corona [41]. The research into improving the interactions with this barrier also suggest some of the most customizable approaches are attractive for further investigation. For instance, utilizing first pass interactions with the plasma proteome to aid in tailoring polymer selection to specific patient application [42]. It is through this innovative approach to “work with” rather than “overcome” biological barriers for nanomedicines that we envisage significant advances will be achieved [43].

### 2.2. Hemolysis

Red blood cells (erythrocytes) are a major cellular component of the blood, and are vulnerable to damage, particularly when exposed to foreign bodies [44]. The term “hemolysis” describes the process by which damage to red blood cells leads to leakage of the iron-containing protein hemoglobin into the plasma. Some nanoparticles have been shown to be hemolytic [45], but also adsorb some of the released hemoglobin and/or adhere to cell debris. This in turn increases the possibilities of clearance by macrophages via scavenger receptors and phosphatidylserine-mediated phagocytosis [44,46,47,48]. Severe hemolysis may lead to life threating conditions, such as hemolytic anemia, jaundice and renal failure. Thus, evaluation of hemolytic activity is a critical aspect of pre-clinical evaluation of polymeric nanomedicines. Many studies have evaluated hemolytic behavior of nanomaterials, such as hard nanoparticles [49,50], soft nanoparticles [51], lipid nanoparticles [52], dendrimers [53], and hyperbranched polymers [54,55] through a standard approach based on spectrophotometric detection of hemoglobin after incubating testing samples with blood [56,57,58,59,60,61,62,63]. However, there is no consensus on a standard hemolysis assay, because of the variations in assay protocols: the blood source (human or animal) and type (whole blood or purified erythrocyte), the incubation time and even the absorbance wavelength measured. Even differences in centrifugation speeds and times can lead to disparate results [64]. Therefore, clear guidelines should be established for evaluation of nanoparticle hemolytic activity before they are approved for intravenous administration, similar to that suggested for standardization of nanomaterial property characterization in bio-nano science [65].

While many nanomedicine systems employ strategies to prevent interaction with red blood cells, recent investigations have looked at utilizing the innate biodistribution and bio-inert properties of these cells. A very promising strategy has described the apparent ability for nanoparticles to “hitchhike” onto red blood cells to improve organ distribution, where the blood cells offer a direct passage to organs of interest [66]. Such an approach offers significantly improved delivery of high-yield therapeutics (nanomedicines) into diseased tissues and is likely to become a hot area of interest into the future.

## 3. Biological Fate of Nanomedicines

While nanomedicines are typically developed to have an effect on, or interact with, specific tissues in the body, they also have defined physical characteristics that dictate their biodistribution and subsequent clearance from the body. Pharmacokinetic and biodistribution behavior is important for tuning the overall therapeutic window of a nanomedicine, in addition to potential off-target effects that might be prominent upon administration [23]. This property is typically tuned to achieve the greatest effect from the added therapeutic cargo. The clearance of the nanomedicines might be related to degradation, destabilization or removal of small polymer components as a result of metabolism. It is important to recognize that this clearance provides insight into the physiological half-life of the nanocarrier itself, rather than the cargo. 

### 3.1. Biodistribution

Upon intravenous administration, nanomedicines distribute throughout the circulatory system and subsequently proceed to compartmentalize in different tissues. The rate of compartmentalization (due to both distribution and clearance) is dependent on the biophysical properties of the polymeric materials and can be described by a number of pharmacokinetic models [67,68]. It is expected that the plasma concentration of nanomedicines would decrease with time due to elimination and excretion via the liver and kidneys, and partitioning and diffusion into tissue. Distribution into major organs such as the liver, spleen, and kidneys are generally due to clearance mechanisms [69] and for a non-fouling polymer such as PEG, is predominantly driven by size and size distribution [70,71]. 

Numerous research groups have attempted to delineate defined cut-off values for organ distribution and this might be somewhat predictable for hard particles, where the radius of hydration (R_H_) is defined and can be correlated to particular sized fenestrations or pores within clearance organs [72]. However, soft particles offer significant challenges in delineating well-defined boundaries and limits for predicting accumulation and clearance owing to their non-rigid size and conformation [73]. The effect of this parameter is discussed in more detail below in the discussion on clearance mechanisms. The various factors that dictate opsonization and interaction with blood components has direct bearing on the biodistribution that is observed. Size, surface chemistry, functionality, topology, and morphology of particles can all play an important role. Likewise, the change in these properties following adsorption of blood proteins will also dictate biodistribution and ultimate clearance routes.

### 3.2. Clearance Pathways

Another important factor to consider is the ultimate clearance of polymeric nanomaterials. As described in previous section, the clearance pathways of particles are dictated by the biophysical properties of the polymer nanoparticles. For example, in the hypothetical case of polymeric materials that are non-fouling and charge-neutral (e.g., hydrated particles constructed from monomers such as PEG), size will dictate the clearance route. Particles exhibiting a size range of < 10 nm will be excreted renally, while hepatic and splenic clearance is typically important for particles < 500 nm through either macrophage clearance or splenic fenestration. However, it has been well demonstrated that hard particles exhibit much clearer size cut-off for a particular clearance route, compared to soft particles where shape deformation can play a role in the clearance observed in filtering organs [74,75]. This is due to potential conformational changes in the soft materials that allow better translocation across membranes (e.g., glomerular filtration) due to chain reputation. This was elegantly demonstrated by Szoka et al. for dendritic structures, where dendritic materials were able to be excreted via the kidneys at a much higher molecular weight than that for PEG [76]. This was further demonstrated for branched polymers [77] that underwent kidney clearance when exhibiting an R_H_ much larger than that expected for renal excretion [78]. 

For the majority of nanoparticle delivery systems, fouling of the surface with proteins invariably occurs. This will ultimately dictate how the nanomedicine will be cleared and limit concentrations of the material in the blood. Significant fouling of small particles can prevent their rapid renal filtration and subsequently enhance circulation (or modulate the mode of clearance), discussed in more detail by Alexis et al. [79]. For the majority of nanoparticle systems, liver accumulation is the major function that occurs following intravenous injection and thus is the major barrier to tumor accumulation of nanomedicines. By far the most prominent mechanism of liver accumulation of foreign particles is phagocytosis by Kupffer cells [80]. These cells reside in the sinusoidal lumen and account for the majority of macrophages in the body. Nanomedicines are also able to pass through the fenestrae along the endothelial wall and become lodged within the perisinusoidal space or interact with hepatocytes. Such processes are ultimately controlled by the biophysical properties of the nanomedicines. Therefore, design parameters of the nanomaterials can significantly modulate the clearance pathway of nanomedicines in a tunable manner.

Given the importance of surface chemistry on the biodistribution and clearance of nanomedicines, recent interest has focused on developing multistep nanomedicine systems that can take advantage of multiple mechanisms for tumor accumulation. It is well established that the EPR effect plays some role (and in many cases the major role) in nanomedicine accumulation in tumor tissue [81]. This is often improved by incorporating targeting ligands towards tumor cell markers that allow for enhanced association with the tumor tissue (these processes are described in greater detail below). One issue with coating nanoparticles with proteins (e.g., antibodies, antibody fragments etc.) is that the injected nanoparticle no longer retains the stealthy characteristics of the nanomedicine (e.g., PEGylated structures), and recognition by the immune system following intravenous injection is exacerbated [82]. To overcome this, new approaches are being developed that utilize a “pre-targeting’ method that is well established in antibody molecular imaging. Here, the targeted system (e.g., antibody) is injected into the patient (or animal) and allowed to distribute and accumulate depending on the biophysical characteristics of the molecule. Following a defined period of time depending on the known pharmacokinetics of the molecule, a second molecule is injected that will have fast clearance, unless it undergoes a chemical reaction with the initial probe molecule. By incorporating imaging modalities on the second molecule, signal-to-noise ratios can be significantly enhanced owing to the much lower background signal present [83,84,85]. By utilizing bio-orthogonal chemistry approaches, this technique can also be translated to therapeutic delivery and offers exciting new methods for improving nanomedicine efficacy, while decreasing off-target effects [86,87]. It is expected that such methodology and approaches will become more popular in nanomedicine therapeutics into the future. 

## 4. Gathering at the Tumor Site

**Figure 3:**
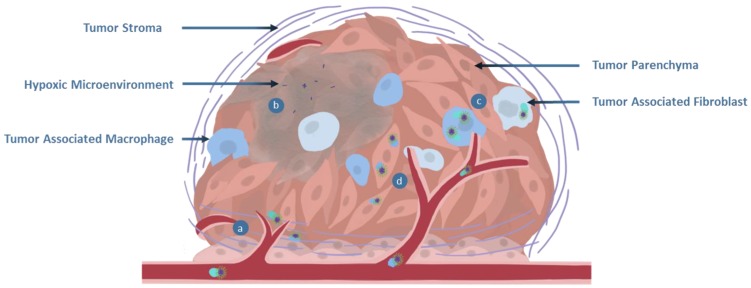
Obstacles associated with tumor accumulation: (**a**) Poor diffusion through tumor stroma; (**b**) premature exposure to delivery stimulus in a hypoxic microenvironment; (**c**) uptake by tumor associated cell populations (e.g., fibroblasts); and (**d**) nanoparticle uptake only within nearby target cells rather than homogeneously throughout the malignant mass.

If a polymeric material evades the immune system [10], transits to the tumor site via convection [88], and opsonization has not rendered it inert [16], the next series of barriers arises when the nanoparticles are required to translocate the vascular endothelium and accumulate within tumor tissue. This is compounded by a number of different biological parameters, including the degree of vascularization, positioning of structures within the tumor volume, blood flow through vessel architectures, and integrity of the endothelial barrier [16,89,90]. Extravasation is critical for polymer transfer from circulation into the tumor tissue. This can occur through a number of mechanisms, including diffusional mechanisms [91], passing through fenestrations [90], transcytosing through endothelial cells [92], or other means [88,93]. A schematic showing some major components of the tumor environment is shown in Figure 3.

### 4.1. Extravasation

#### 4.1.1. Impact of Vessel Architecture

An initial consideration governing whether a nanomaterial will reach the interior of a solid tumor is the distribution and complexity of the vessel architectures endogenous to the cancerous tissue. Processes associated with cancer biology such as neo-angiogenesis and vascular mimicry allow for high variability and complexity of associated vascular [94,95,96]. The extent to which tumor tissue is vascularized and the perfusion of those vessels directly correlates with nanoparticle extravasation [93,95]. Tied to the disease stage, volume of the mass and metabolic needs of the tumor, microvasculature generated via neo-angiogenic processes often comprises a suite of abnormal features. This includes factors such as variable vessel thickness, disorganized vascular branching, poor basement membrane integrity, irregular endothelial cell phenotype, and functional shunting [88,89,97]. The permeability of vessels produced through neo-angiogenesis are usually a key determinant as to whether vessel architecture plays a significant role in the EPR effect. This impacts nanomedicine accumulation as this phenomenon typically results in vasculature with the best access to the tumor interior [94,95,98]. Further, angiogenesis at the tumor site is in part responsible for the development of the complex tumor microenvironments [88]. Comparisons of xenograft tumors arising from different cancer cell lines have found that specific examples possess a higher propensity for the development of vascularity when used to establish tumor models than do others [93,94,96,99]. This indicates that in the preclinical space, tumor model selection holds great sway over the behavior of nanomedicines relying on the EPR effect. This is difficult to account for when considering clinical translation. The role of vascular endothelial growth factor (VEGF) in these processes has resulted in it becoming an attractive target for accumulation at the tumor site [88,100]. Co-delivery of vessel normalizing drugs, vasodilators, and remodeling of the cancer-associated vasculature have also become prominent approaches to improving the accumulation of nanomaterials at the tumor site, with varying levels of success reported [101,102,103].

The ability to control or predict extravasation in vessel architectures has become an important point of study. This is because drug delivery at undesired locations can cause vascular collapse and subsequent shunting, thus reducing the accessibility of the tumor interior to the remaining polymer in circulation [88]. As with many of the biological barriers discussed, understanding the formation of vascular architectures in tumors and the causal mechanisms that result in extravasation of polymeric materials will improve the probability of nanomedicines translating into the clinical sphere.

#### 4.1.2. Role of Endothelial Integrity

The structural features that govern permeability of vascular architectures and thus whether tumor tissue may be imbued via the EPR effect, are static and dynamic openings in the endothelial barrier, termed fenestrations [104]. In particular, neo-angiogenic vessels often possess variable permeability due to their structurally unreliable production resulting in the distribution of fenestrations throughout tumor vasculature being similarly chaotic [105]. Combined with flow rate within a particular vesicular location, the size and frequency of fenestrations contribute to extravasation efficiency, particularly with respect to nanomedicine size [106]. These vascular openings are under dynamic regulation and the resulting venting behaviors represent an important means by which larger materials may translocate the endothelial barrier [90]. The distribution and modulation of fenestrations are partially responsible for uneven penetration of nanomedicines into the tumor tissue [107]. While the spatial regulation and tumor phenotype of endothelial fenestrations and their venting behaviors remain elusive. It can be inferred that if a region bearing a higher concentration of fenestrations or increased permeability is associated with tumor parenchyma then drug delivery will be significantly improved over an area associated with the stroma. Given their decisive role in dictating size-dependent escape from vasculature, fenestrations consequently govern the concentration and distribution of nanomaterials that may obtain access to the tumor volume and thus exert control over accumulation. An interesting point to note is that hard particles have been found to improve their own tumor accumulation through damaging the endothelial barrier at the expense of inducing metastasis [108]. However, the applicability of this finding to soft materials such as polymeric systems remains dubious.

#### 4.1.3. Emerging Tools for Imaging and Overcoming the Endothelial Barrier

Molecular imaging techniques offer unique insight into the various biological processes that a nanomaterial may undergo following injection into animals. Importantly, the imaging can inform on both spatial distribution of nanomedicines, as well as provide functional information on bio–nano interactions [109,110]. Magnetic resonance imaging (MRI) is among the best-established techniques for understanding the underlying biology of vascular architecture and endothelial integrity. This imaging modality benefits from a suite of techniques and contrast agents that have been developed. Numerous ligands for MRI have already been established, and are commercially available for incorporation into nanomaterials through chemical handles [111], encapsulation [112], or conjugation of chelation motifs [113]. Similarly, dynamic positron emission tomography (PET) imaging also provides a unique insight into accumulation within the tumor mass through uniquely quantitative data. Strategies/agents for incorporating radiometals with appropriate half-lives are also well established [114]. While useful and clinically relevant, these techniques generally lack either the spatial or the temporal resolution for examining the biological interactions of intravenously administered nanomedicines. Traditional fluorescence imaging also lacks the spatial resolution required to image extravasation processes at work. However, the inclusion of fluorophores in the composition of polymeric constructs allows for correlation of in vivo imaging with ex vivo microscopy of tumors and tissues, with high spatial sensitivity. Analysis of biological samples ex vivo has become increasingly commonplace for a variety of investigative purposes in terms of the distribution of materials with respect to blood vessels. For instance, confocal microscopy of sectioned tumors [100,115] and light sheet microscopy of cleared tumors [91] and organs [116], represent a means by which intra-tissue distribution of materials and their relationship with vasculature can be studied at a tissue or cellular level. Unfortunately, this resolution comes at the expense of the ability to capture dynamic behaviors with reliability. Microfluidics offer one of the only means for mimicking vascular conditions and controlling specific factors such as flow rate experimentally [117,118]. While excellent advances have been made [119], much like other in vitro methods, this approach often suffers from bias and lack of translational relevance on account of its heavily artificial nature. 

Emerging imaging modalities may be better equipped to answer the foundational questions regarding dynamic nanomaterial behaviors at the endothelial barrier in vivo; for instance, photoacoustic imaging and intravital microscopy (IVM). As demonstrated by Matsumoto et al. (2016) [90], IVM is well suited for capturing processes associated with the endothelial barrier, being able to visualize liposomes engaging in extravasation processes sequestered into size-limited mechanisms. The use of IVM and similar techniques has become more commonplace owing to its ability to image dynamic processes and determine pharmacokinetics at a cellular level in model animals in real time. Photoacoustic imaging offers a unique means of visualizing vessel architecture and monitoring the flow of blood through the tumor mass, in addition to tracking exogenous probes. This technique can utilize the properties of hemoglobin to provide label free imaging for the examination of oxygen levels and perfusion of the tumor mass. While this modality is still very much in its infancy, efforts to develop photoacoustic probes for monitoring in vivo behaviors of polymeric materials are well under way, with encapsulation of select dyes [120,121] and attachment of quenching molecules [122] being prominent examples. 

In terms of delivering nanomedicines across the endothelial barrier, a number of methods have been developed to improve the rate of extravasation and thus accumulation within the tumor mass. Recent efforts have demonstrated that the EPR effect may be improved through altering blood vessel geometry [102], normalizing vasculature [101], remodeling [103], and vessel pruning [123]. Microbubble cavitation can also be used to disrupt endothelial barriers and increase flow of material from vasculature into the tumor mass [124]. However, given the concerns raised by the recent findings regarding hard particles inducing metastasis through damaging endothelial integrity [108], this solution may not prove clinically viable for applications involving aggressive or metastatic tumors.

### 4.2. Tumor Tissue Distribution

#### 4.2.1. Tumor Stroma and Microenvironments

An important consideration for determining the outcome of therapeutic delivery is the location within the tumor mass where polymeric materials overcome the endothelial barrier. For instance, if the material has greater access to the tumor stroma than parenchyma. Stroma is the site of dense extracellular matrix and tumor associated cell populations, which represent significant obstacles to tumor perfusion. Should a material find itself in this milieu, difficulties may arise in specific accumulation at a site where therapeutic release will prove effective. Given that stroma formation and hypoxia possess key roles in the initiation of neo-angiogenesis, there is an interplay between tumor microenvironments, vessel architecture and endothelial integrity [125]. As with vascular architecture, Sulheim et al. [93] identified that components of the stroma, such as collagen concentration, vary with the cell line used to establish xenograft tumors. The differing levels of fibrous content also contribute to the ability of materials to diffuse through the tumor environments. The density and positioning of blood vessels relative to the parenchyma and microenvironments of the tumor mass are key elements contributing to whether a material will be able to obtain access to a location of therapeutic benefit. Should a material be able to avoid or overcome the stromal barrier, it will then face the microenvironments of the tumor bulk, which may vary drastically in terms of biological content and chemical species present. These factors are particularly pertinent to environmentally responsive polymers as such microenvironments may prematurely trigger release mechanisms such as degradable linker motifs. Hypoxic regions are known to occur in the majority of solid tumors and should be taken into consideration in the design of polymers for nanomedicine applications [126].

The interstitial pressure resulting from microenvironment development also produces a difficult obstacle, with passive diffusion hindered relative to the size, stage, and specifics of each tumor. Larger, poorly vascularized tumors have a tendency to produce pressure gradients that may thwart polymer penetration. This forces the material to remain towards the tumor periphery and leaves them open to discovery by tumor associated macrophages (TAMs). As reported by Lucas et al. [127], endogenous macrophages may possess specific distributions in xenograft models and modulate the pharmacokinetics of drug delivery, altering the behavior and efficacy of materials. The understanding of this interaction is confounded by a complex array of interactions that exist amongst tumor cell populations, which has been well reviewed elsewhere [128].

The combination of these factors with the aforementioned barriers, are likely the source of the commonly cited < 10% of injected dose accumulating within the tumor mass [3,16]. This is dependent on the stromal barriers and internal distribution of materials and thus may yield a lower effective dose to the tumor tissue in certain cases. As noted prior, few studies report the intratumoral distribution of the materials being studied. However, as numerous groups report successful accumulation at the tumor site, improving the distribution, and more importantly the penetration, of nanomedicines has become an important objective in ongoing developments in the field.

#### 4.2.2. Spatial Regulation of Receptor Expression

A further limitation of tumor perfusion specific to accumulation at the tumor site is the expression of target receptors and proteins. This is a specific factor relevant to targeted materials. Although receptor targeted materials commonly outperform those that rely solely on the EPR effect, such designs are not without their own set of hurdles. Even in xenograft tumors, receptor expression is not ubiquitous throughout the mass. Biological stimuli modulating gene expression of the cells during growth and the formation of microenvironments induce heterogeneity within the parenchyma and stroma of the cancerous tissue. For targeted materials, not only do these materials need to be able to penetrate the tumor parenchyma, but also find their target receptor or marker. Assuming that the material encounters its target receptor, a number of the prior barriers (such as opsonization) may have altered the biological identity of the material, potentially inhibiting the binding interactions required to induce cellular internalization [16].

#### 4.2.3. Downstream Influence of Opsonization on Receptor Affinity

**Figure 4:**
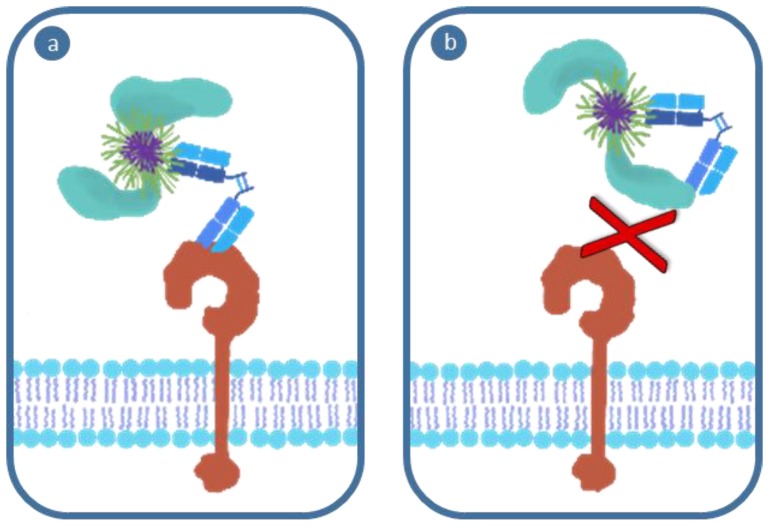
Schematic demonstrating the impact of opsonization on targeting efficiency: (**a**) Desired targeting, wherein binding is maintained; and (**b**) binding hindered by interactions with non-specifically bound protein.

Despite a targeted nanoparticle exhibiting the ability to migrate through the barriers outlined above and reach the target receptor, the complex array of biological modifications to its surface as a result of in situ opsonization may still yet inhibit its functionality (Figure 4). Prior interactions in the bloodstream and the nature of the protein coat acquired throughout the journey may result in a reduction in binding affinity, avidity and accessibility of the targeting motif. These factors can produce non-specific cellular association or reduce the ability of the polymer to interact with the receptor through steric hindrance or corruption of the targeting moiety. Even in the event that the targeting motif is still available for binding, access to endocytic pathways may also be altered should a material become too large for the receptor-mediated pathway of the target (e.g., in the case where opsonins etc. modulate nanomedicine size). While the interactions between physiochemical properties and biological identity are regularly discussed, few studies have explored the impact of these phenomena in detail. As reviewed by Lazarovits et al. [16], these behaviors are dynamic and the outcome unpredictable, although with a wider array of systematic studies and improved reporting standards to facilitate meta-analysis [43,65], our ability to ensure targeting fidelity will improve. 

#### 4.2.4. Current and Emerging Tools for Improving Penetration of the Tumor Mass

Several methods have been suggested to improve transit of materials through the tumor mass, with numerous options available to alter the structures and conditions within the tissue. These include examples such as remodeling stromal architecture through destruction of collagen [129], inhibiting or modulating processes contingent to microenvironment formation [130], and implementing heat and radiation to allow for limited control over fluid dynamics [131].

Although discussed as one of the barriers to penetrating the tumor mass, one means for improving access to the tumor parenchyma is to target the endogenous macrophage populations. By targeting the tumor associated immune cell population, macrophage shuttling across the tumor stroma may be one of the effective means for transporting materials [132]. However, this may pose issues for environmentally responsive polymers targeted to endosomal compartments, as it is highly likely they will encounter this stimulus within the carrier cell.

A recent review by Sun et al. [133] showed particular sizes, shapes, surface charge, and end-group functionalities have potential to improve intratumoral distribution. Surface architecture has also been indicated as a means to alter tumor penetration of materials [134]. As our understanding of the interplay between such factors and permeation of cancerous tissue has improved, a number of innovations in polymeric design have been developed to help facilitate tumor penetration and overcome the influence of microenvironments. Mechanisms that enhance drug release through polymer disassembly are one such example [135], and offer improved opportunities to personalize nanomedicines. Further, including motifs and architectures that allow for charge- and size-switching of materials have been demonstrated to provide better penetration in xenograft models. For instance, Li et al. [136] demonstrated that through pH-dependent disassembly, a particle designed for improved circulation times could improve penetration into the tumor mass once it encounters acidic tumor microenvironments. Similar observations have been reported for hyperbranched polymers bearing hydrazone-linked chemotherapy drugs, whereby the released drug was able to transit further from the vessel architecture than the associated polymer vehicle [137]. Materials that shed their PEGylated surface have also become a popular design choice for improving the behavior of polymeric materials at the tumor site [138,139]. 

## 5. Cellular Level Interactions and Behaviors

All chemotherapeutics act on an intracellular target [140]. A patient undergoing a common chemotherapy regime is administered with a combination of alkylating agents, anti-metabolites, mitotic inhibitors, topoisomerase inhibitors, and cytotoxic antibiotics [141]. These therapies interfere with microtubule biology, inhibit enzymes involved in deoxyribonucleic acid (DNA) synthesis, replication, and repair, and generate breaks in DNA, all of which are located in specific cellular compartments such as the cytosol and nucleus [142]. Emerging therapeutic strategies like protein, small interfering ribonucleic acid (siRNA) and clustered regularly-interspaced short palindromic repeats/Cas9 gene engineering (CRISPR/Cas9) also operate on targets in specific intracellular locations [143]. Therefore, to generate their anticancer effect, the therapeutic must migrate to this site of action. 

Once accumulation and penetration throughout the tumor tissue has occurred, the therapeutic must surpass a series of cellular barriers to migrate to the necessary subcellular organelle [144]. These intracellular processes required are determined by the properties of the specific therapeutic and its mechanism of action [145]. Small molecule chemotherapeutics can partly accumulate in the necessary subcellular location through passive diffusion, whereas membrane impermeable biological therapeutics require active transport [144]. In general, nanomedicines are developed such that the physicochemical properties of the encapsulated therapeutic would have no significant effect on the biodistribution and tumor localization of the nanomedicine itself. In contrast, as the intracellular barriers required vary between therapeutics, a therapeutic specific nanoparticle design is likely to be required to navigate the intracellular processes hindering subcellular localization. These include strategies to enhance internalization, generate escape from or bypass the endocytosis pathway, and facilitate intracellular trafficking [144]. Regardless of the properties of the therapeutic, improving subcellular delivery invariably leads to an improvement in efficacy [146,147,148]. It is also an important strategy to overcome the downstream complications that are encountered when treating multidrug-resistant forms of cancer [149].

### 5.1. Internalization 

Nanoparticle internalization is the first step to subcellular localization. Although both energy-dependent and energy-independent uptake are often observed, nanoparticle internalization through the endocytosis pathway is the most prevalent [150]. There are a myriad of cellular endocytosis mechanisms including caveolae-dependent, clathrin-dependent, clathrin/caveolae-independent, phagocytosis, and pinocytosis, which broadly involve the budding and tethering of the cell membrane into an endocytic vesicle [151]. As internalization varies greatly between both particles and cell-type, understanding nanoparticle internalization pathways is important to optimizing the delivered intracellular dose of the nanoparticle [152].

#### 5.1.1. Nanoparticle Properties That Influence Cell Internalization

As highlighted in recent reviews, the physicochemical properties of a nanoparticle including size, charge, and surface chemistry, as well as the addition of targeting ligands, impart great influence on the cell association and internalization [150,153,154]. In particular, a positive charge enhances cell association through the interaction with the negatively-charged phospholipid bilayer of the cell membrane [150,155]. While an improved cell association can result in enhanced internalization, these two processes are not always intertwined. As such, investigations into the effect of the nanoparticle’s physicochemical properties on cell internalization are necessary [156]. An emerging study by Caruso and coworkers [157] highlights the complex interplay between nanoparticle physicochemical properties and cell internalization. In the study, alterations to the size and shape of poly(ethylene glycol) capsules functionalized with bispecific antibodies influenced cell internalization, while a negligible difference of cell association was maintained. Chen and coworkers [158] present another interesting observation that the fluorous substitution of alkanes enhanced the internalization of branched polyethylenimine (grafted with hydrophobic substituents) and bovine serum albumin nanoassemblies. This could present and exciting avenue to enhance particle internalization. 

Modifying the nanoparticle design; however, must be made with careful consideration, as enhancing association and internalization can also increase non-specific uptake and alter biodistribution (as discussed in Section 3.2). For example, the addition of a stealthy PEG shell reduces non-specific uptake by the immune system, but also reduces target cell association and internalization [159,160,161]. While a balanced approach of enhanced target cell internalization with not too much non-specific uptake could be taken, a strategy to tune the nanoparticle design to switch on internalization at the target site is likely to be most effective. Current strategies include incorporating a detachable corona and modulating particle charge inside the acidic tumor microenvironment (as discussed in Section 4.2.4) [162,163,164].

A different strategy to avoid the endocytosis pathway and gain intracellular access is by fusion with, or translocation through the plasma membrane [165]. While this strategy has not been demonstrated for polymeric nanoparticles; inorganic nanoparticles [166], liposomes [167], cell penetrating peptides [168], and hybrid particle nanoassemblies [169,170] have all been observed to enter cells by fusion or translocation. An important demonstration of this strategy was reported recently using nanoassemblies of arginine functionalized gold nanoparticles self-assembled with oligo(glutamate) tagged proteins capable of rapid fusion with the plasma membrane [169]. In order for a particle capable of fusion to be used for cancer nanomedicine, selective fusion with the cancer, and not host cells, is needed for safe and targeted treatment. The acidity of the tumor microenvironment, around pH 6.8–6.4, provides an opportunity to distinguish between host and cancer cells [171]. It is also possible to control fusogenic capabilities with a stimuli-responsive particle corona, as discussed for control of internalization above [172]. 

All of these nanoparticle modification strategies offer the opportunity to capitalize on chemists’ ability to design complex materials based on increased biological knowledge of the tumor microenvironment. Much of the literature has focused on nanoparticle cell association; however, this can be different to cell internalization. An additional focus on nanoparticle internalization and the continued understanding of the complex tumor microenvironment will enable the rational design nanomedicines with efficient and selective cellular internalization [152].

#### 5.1.2. Tools to Understand Cellular Internalization and Limitations of In Vitro Models

When investigating internalization, it is important to distinguish between associated and internalized material, since many techniques are used erroneously to study these phenomena ubiquitously including fluorescent activated cell sorting (FACS) [152]. Investigating internalization can be achieved through confocal microscopy and electron microscopy, but are generally low-throughput, time-intensive, and sample-dependent [150]. Confocal microscopy, either in fixed and live cells, allows the location of nanoparticles to be observed visually by co-staining the cell membrane and various organelles [156]. While viable for inorganic nanoparticles, measuring internalization through electron microscopy can be complicated for polymeric nanoparticles as they are difficult to distinguish from their biological environment, due to possessing components with similar electron density [173,174]. Improved contrast can be achieved through the staining with heavy metal salts; however, not all polymeric nanoparticles carry functions groups that respond to the staining [174]. Doping the particle with electron dense components such as inorganic nanoparticles or quantum dots is also another method explored to enhance contrast [4,175]. A more elegant strategy that could be applied to the study of polymeric nanoparticles would be to enhance the electron density of the particles through immunolabeling with antibody-gold conjugates or ascorbate peroxidase (APEX) [176,177,178]. 

High-throughput methods to quantify internalization are the most beneficial, as they logistically enable the screening of large libraries of both modifications to the nanomedicine and biological variables. As a result, methods have been developed to enable the high throughput flow cytometry measurements to correspond to internalized nanoparticles [179,180,181]. Gottstein and coworkers [179] developed a method to correct flow cytometry data that involved the determination of the ratio of associated vs internalized nanoparticles by confocal microscopy, then after correlating the nanoparticle fluorescence in confocal microscopy to flow cytometry, the application of a correction factor to the flow cytometry data. The implementation of an internalization mask to imaging flow cytometry, that only includes fluorescence located within an area defined by the fluorescently-labeled cell membrane, has also been used to measure internalization in a high throughput manner [157]. Another strategy involves the use of sensors to probe the difference between internalized and associated nanoparticles. The specific hybridization internalization probe (SHIP) and the fluorescent click internalization sensors quench fluorescence from surface-bound material, this allows a correlation between the total fluorescence and percentage of internalized nanoparticles to be determined [180,181]. Other methods of extracellular quenching are trypan blue and acid washing; however, these methods are limited for applications that require other surface-bound fluorescence such as phenotyping [181]. The inhibition of endocytosis through incubation at low temperature can also be used to provide an indication of the particles associated with the cell [180].

The in vitro assays typically used to investigate internalization are not sufficient to understand the internalization of nanocarriers in the complex tumor environments. Internalization assays are typically performed on cell culture dishes under static conditions and, as a consequence, are impacted by nanoparticle sedimentation and a lack of ligand-binding competition [182,183,184]. The study of nanoparticle cell association and internalization through an in vivo model would be the most physiologically accurate, but currently no standardized assay is available owing to the complexity of the system under study. A possible approach could be to sort through the complex array of cell types present in a tumor using fluorescence-activated cell sorting, and then probe internalization using the SHIP and fluorescent click internalization sensors mentioned above [150]. 

### 5.2. Endosomal Escape 

Generating endosomal escape is critical for nanoparticles internalized via endocytosis. After internalization into an endocytic vesicle, the nanoparticle will travel down the endocytosis pathway to either mature into a late endosome and accumulate in a lysosome or be exocytosed out the cell [165]. Remaining in the lysosome is highly detrimental for a nanomedicine, especially for biological therapeutics, as the therapeutic remains segregated from the intracellular environment and is degraded by the acidic and enzyme-rich environment of the lysosome [185]. Engineering materials to overcome this bottleneck is a necessary step for efficient intracellular delivery. Detailed accounts of the current strategies and materials to generate endosomal escape, as well as the methods to determine escape, are covered in previous reviews [165,186].

#### 5.2.1. Engineering Materials to Escape the Endosome

One strategy to enhance the endosomal escape of a membrane impermeable therapeutic is to engineer the nanoparticle design to respond to stimuli endogenous to the endosomal pathway to generate a response that disrupts the integrity of the endosome. The polymeric nanoparticles investigated for this approach are typically pH-responsive materials, which respond to the pH decrease during the maturation of the endosome, from a physiological pH of 7.4 down to pH 5.0 of the lysosome. The generation of endosomal escape is postulated to be through lysing of the endosome through mechanical stress from particle swelling or by rendering the endosomes leaky through membrane disruption and the formation of pores [186]. A prominent example is the pH-responsive core shell particle, composed of a poly(2-(diethylamino)ethyl methacrylate) core and poly(aminoethyl methacrylate) shell that was crosslinked with poly(ethylene glycol) dimethacrylate, demonstrated to induce efficient endosomal escape of calcein compared to a pH-insensitive control particle [187]. In another study Shen and coworkers observed that increasing the amount of a poly(2-(dimethylamino)ethyl methacrylate-*co*-propylacrylic acid-*co*-butyl methacrylate in a blend particle with poly(lactide-*co*-glycolide) enhanced the endosomal escape of calcein [188]. This was thought to be by pH-dependent membrane interaction. A major limitation of the current pH-responsive nanoparticle strategies based on membrane interaction is the size of cargo that can escape the endosome, as there are numerous reports of the escape of small cargo but limited examples of large cargo [189,190,191]. Further understanding on strategies to facilitate the endosomal escape of large material are needed to enable the efficient intracellular delivery of biological therapeutics. While pH is an obvious choice of stimuli, additional considerations are needed to use pH-responsive materials to generate endosomal escape in cancer cells. If the response of the nanomaterial in the pH range ~6.8–6.4 is faster than the rate of cell internalization, then activation will occur in the slightly acidic tumor microenvironment rather than inside the endosome, leading to poor efficiency of the nanomedicine [192]. 

Nanoparticles that respond to stimuli exogenous to the endosomal pathway has also been investigated as a method to generate endosomal escape [193]. Photochemical internalization (PCI) investigates the use of light to generate endosome escape. In these systems, upon the excitation by light, a photosensitizer molecule in the vicinity of the endosomal membrane generates reactive oxygen species that disrupts the membrane to facilitate endosomal escape [194,195]. Using this approach, photosensitizer molecules have been attached to nanoparticles and have been shown to induce the transition from punctate to diffuse fluorescence of labeled dextran. This approach also showed the enhancement of various endpoint assays after radiation [196]. For example, a tertiary complex of a phthalocyanine-centered second-generation aryl ether dendrimer, a dimerized cationic peptide, and plasmid DNA was observed to facilitate the endosomal escape of 10–15 kDa dextran upon stimulation of 400–700 nm light [197]. The depth of light penetration and the potential for toxicity are limitations of this approach. A recent study by Nogués and coworkers also indicates that there is a size threshold for cargo that can escape the endosome using PCI [194]. 

**Figure 5:**
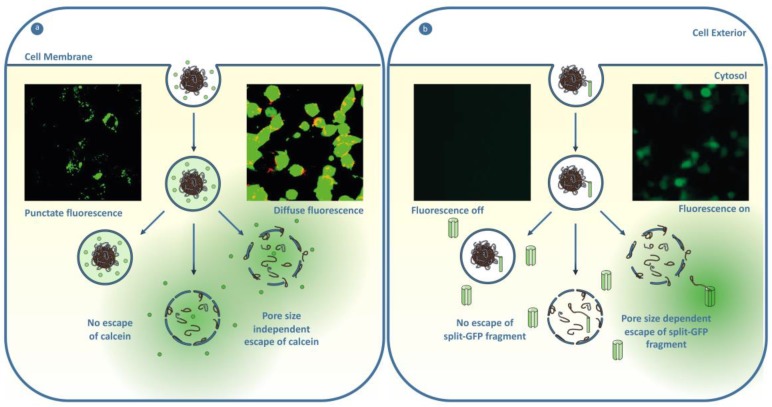
Schematic representation of the current and emerging methods to determine endosomal escape. (**a**) The calcein assay determines endosome escape via the transition from punctate to diffuse fluorescence throughout the whole cell. (**b**) The split-green fluorescent protein (GFP) assay generates a fluorescent signal after endosomal escape and reconstitution of the GFP-strand with the cytosolically-localized GFP. The split-GFP assay allows the endosomal escape of large therapeutics to be monitored, in contrast to the calcein assay, which can only represent the escape of small molecules. Adapted with permission from [187,203]. Copyright 2007, American Chemical Society. Copyright 2010, John Wiley and Sons.

Another method for endosomal escape of cargo is to modify the encapsulated therapeutic with the ability to escape the endosome. This has been demonstrated through the covalent attachment of cell-penetrating peptides (CPP) or the remodeling of a protein’s physicochemical properties that can deliver the therapeutic to the cytosol by the formation of transient pores or through direct translocation. The covalent attachment of the cyclic trans-activator of transcription (cTAT) cell-penetrating peptide has been shown to facilitate the delivery of the attached green fluorescent protein (GFP). While this is a great example of delivering a large protein, the high concentration of 150 μM of the GFP-cTAT conjugate needed is likely to be too high to work as a nanoparticle cargo [198]. Endosomal escape at lower concentrations were demonstrated by Dowdy and coworkers [199], who observed that GFP fluorescence of the split-GFP assay increased by 14-fold when using 45 μM of GFP_11_-TAT-P6-GFWFG (GFP fragment conjugated to trans-activator of transcription (TAT) modified by a polyethylene glycol spacer of six repeat units and a GFWFG peptide). While covalent approaches are achieving some success, there is the potential to disrupt the activity of the therapeutic delivered, and currently retention of function has not been demonstrated. Incorporating a cleavable group that responds to pH, redox potential, or enzymes could be a method to remove the covalent modification to regenerate the functional protein. The modification of the surface properties of GFP with bio-reducible esters was found to enhance the cytosolic delivery at 15 μM by enabling the translocation, and once inside the cell the modification was removed through intracellular esterases [200]. However, demonstration that activity was retained was not explored. Delivery of functional enzymes has been demonstrated by Schepartz and Wagner [201,202]. Wagner, Lächelt and coworkers developed a three-arm cationic star succinoyl tetraethylenepentamine conjugated with a tetraethylene glycol linker by a pH-responsive aminated methyl maleic anhydride bond [201]. The protein star conjugate showed some diffuse fluorescence of enhanced green fluorescent protein (eGFP) at a 1 μM of protein, and was demonstrated to induce apoptosis through the similar delivery of RNase A star conjugate. Endosome escape at 1 μM was indicated by Schepartz and coworkers using a zinc finger protein modified by arginine motif 5.3 (ZF5.3), as indicated by the fluorescence correlation spectroscopy (FCS), the glucocorticoid-induced eGFP translocation (GIGT) assay, and increased cytosolic activity of the APEX conjugates (refer to Section 5.2.2 for more details of FCS and GIGT assay) [202]. However, the confocal images suggest low levels of escape, as fluorescence is mostly punctate with some areas of diffuse fluorescence. Interestingly, enzyme ZF5.3 conjugates were demonstrated to retain activity without cleaving the CPP. The covalent modification of the therapeutic approach requires the encapsulation and release to be in the endosome of the target cell or tumor microenvironment for targeted treatment (Section 4.2.4). Loading into a polymeric nanocarrier could be used for this, but to our knowledge this has not been investigated yet [Figure 5]. 

Developing materials to facilitate the endosomal escape of nanomedicines is still a developing field and a generalizable method to obtain escape for a wide array of therapeutics remains unanswered. An increased focus on understanding endosome escape and the mechanisms by which escape occurs will enable the rational design of future nanomedicines. 

#### 5.2.2. Tools to Understand Endosomal Escape

The development and implementation of robust and standardized assays are needed in order to develop efficient endosomal escape materials. Endpoint assays, such as transfection efficiency, are commonplace when investigating endosomal escape [186]. While a useful indirect measurement of escape, they limit the formation of specific and generalizable knowledge on endosomal escape, as generally a particle’s performance in an endpoint assay is not easily uncoupled from other variables such as loading, uptake, and subcellular trafficking. The absence of colocalization with endosomal and lysosomal markers is also another commonly reported method, but this does not necessarily indicate endosomal escape, if the fluorescence remains punctate [165]. A more robust strategy to measure endosomal escape is the calcein assay. The calcein assay visualizes calcein, a membrane-impermeable dye as punctate fluorescence when entrapped, and as diffuse fluorescence throughout the whole cell after endosomal escape [187]. However, the calcein assay is limited by techniques to quantify the results and that it can only represent the escape of a small molecule [191]. When trying to deliver biological molecules, it is important for an endosomal escape assay to be able to report on the escape of larger sized cargo, as larger size material has been observed to remain co-localized with lysosomal markers while showing diffuse calcein [191]. The endosomal escape of large cargo can be probed via a variety of emerging techniques that aim to better distinguish endosomal from cytosolic fluorescence by measuring changes in fluorescence, or through the interactions of the cargo with a cytosolically-located enzyme (for more detail the reader is referred to the following review: [204]). Fluorescence correlation spectroscopy measures the enhanced diffusion of a dye-labeled material of interest upon liberation from the confinement of the endosome [202,205]. Protein-fragment complementation assays such as the split-GFP assay can also be used to measure endosomal escape [206]. In this assay, a split-GFP fragment is conjugated to the cargo or nanoparticle, and upon endosomal escape, reconstitution with the rest of cytosolically-localized GFP generates a fluorescent signal. Another assay that enables the probing of the endosomal escape of biologically-relevant cargo is the glucocorticoid-induced eGFP translocation assay (GIGT) [207]. When located in the cytoplasm, the glucocorticoid receptor (GR) ligand dexamethasone (Dex) induces the translocation of the glucocorticoid receptor eGFP (GR-eGFP) conjugate from the cytoplasm to the nucleus. The GIGT assay then measures the nuclear-vs-cytoplasmic fluorescence that indicates the amount of glucocorticoid-induced eGFP translocation induced by the endosomal escape of Dex tagged cargo. A potential limitation of the split-GFP and the GIGT assay is if attaching the material of interest disrupts the ability to bind with the protein for the assay to report, resulting in the underrepresentation of escape [208]. On the other hand, as the delivery of functional materials to the cytosol is the aim then probing the endosomal escape of a functional material might be more beneficial than probing total endosomal escape. 

The emergence of standardized and quantified endosomal escape assays, which are capable of representing the escape of a wide range of therapeutics, will enable further understanding into how nanoparticle structure and biological variations influence endosomal escape. Optimizing this process will enable higher nanoparticle doses to reach the target region of the cell and thus improve therapeutic efficacy.

### 5.3. Trafficking to Subcellular Locations

After endosomal escape, the therapeutics that require organelle localization must be trafficked from the cytosol to other subcellular compartments. In some cases, such as a doxorubicin delivery to the nucleus, this occurs through passive diffusion. For numerous therapeutics, however, active delivery to these subcellular organelles is required and is a requisite component when considering design of a suitable nanocarrier. This importance is best demonstrated by Escande and coworkers who observed that less than 1% of plasmid DNA injected directly into the cytoplasm was trafficked to the nucleus [209]. This factor dictates in a large sense the efficacy of many therapeutic systems (in particular gene therapies). A detailed review of strategies to improve subcellular localization are covered in previous reviews [143,145].

#### 5.3.1. Trafficking to the Nucleus

The ability to traffic a therapeutic from the cytosol to the nucleus is initially determined by the size of the therapeutic being delivered. Transport through nucleus’ double phospholipid bilayer is mediated through the nuclear pore complex, which allows passive diffusion of material of less than 45 kDa or ~ 9 nm in size [210]. If the therapeutic delivered is ~ 9 nm in size or less, then upon cytosolic delivery the passive accumulation into the nucleus will occur. Therapeutics above 9 nm in size require an active delivery method. The conjugation of a nuclear localization signal (NLS) to the therapeutic enables access to the cell’s nuclear import machinery. Through complexing with importin α/β, just importin β, or by an importin α/β-independent pathway, the delivery of larger molecules into the nucleus is achieved [211,212,213]. This was well demonstrated by Rotello and coworkers who enhanced the nuclear localization of eGFP through the conjugation with an NLS [211]. Once delivered to the cytosol by nanoparticle-stabilized nanocapsules, fluorescence was demonstrated to migrate from the cytosol to the nucleus. Five NLSs were tested, including sequences derived from the EGL-13 transcription factor, the simian virus 40 (SV40), the c-Myc protein, the nucleoplasmin protein (NLP) and the tus protein (TUS), with c-Myc and NLP observed to enhance the transition from nuclear to cytosolic fluorescence the most. Nuclear localization of eGFP was not observed without the NLS and for eGFP-NLS under adenosine triphosphate (ATP) depleted conditions, indicating that the enhanced localization was a result of accessing the nuclear import machinery. A similar approach has also been used to deliver oligo(glutamate) tagged Cas9 protein to knock out a gene in RAW264.7 macrophage cell line [214]. 

The addition of an NLS; however, is not always linked to enhanced nuclear transport [215]. As a result, the addition of an NLS must be optimized on a case-by-case basis, as the type and number of conjugated nuclear localization sequences all influence the efficiency of nuclear transport [216,217]. The size of the material imported must also be considered; however, the reported upper threshold size has varied between studies [147,212,216] and highlights the lack of sufficient knowledge about this important transport mechanism. Another method to facilitate nuclear import is via cationic polymers, suggested to enhance localization through the permeabilization of the nuclear membrane. However, nuclear permeabilization is linked to high cytotoxicity [218,219].

#### 5.3.2. Trafficking to the Mitochondria

Mitochondrotropic molecules and mitochondria targeting signals allow trafficking through the mitochondria’s phospholipid bilayer from the cytosol [220]. Attaching a mitochondrotropic molecule to the nanoparticle or cargo facilitates mitochondria targeting via the attraction between the delocalized positive charge of the amphiphilic mitochondrotropic and the negative potential of the mitochondrial membrane [143]. Recently this approach was used by Kempe and coworkers, who observed that the addition of cyanine 5 dye molecule to a carboxylated N-acylated poly(amino ester) based comb polymer resulted in the passive diffusion through the plasma membrane and colocalization with the mitochondria through confocal microscopy [221]. Interestingly, the mitochondria targeting was facilitated despite the overall negative charge of the polymer. Further investigations into the structure–property relationship of the comb polymer revealed that modifications to the cyanine 5 dye, the carboxylic acid pendent groups, and the polymer size altered the polymer internalization and prevented the subsequent localization to the mitochondria. Conjugation of mitochondria targeting signals also enables the transport of material into the mitochondria via mitochondria import machinery [222,223]. While mitochondria-targeting signals have been used for hard nanoparticles, this approach is yet to be applied to soft nanomaterials [223,224,225].

#### 5.3.3. Emerging Tools for Quantification of Sub-Cellular Localization

There are still limited strategies to achieve sub-cellular quantification. One common technique is by measuring colocalization of fluorescently-labeled nanoparticles or cargo with organelle markers using confocal microscopy and electron microscopy. These techniques, as seen with internalization (Section 5.1.2), have limitations of being low-throughput, time-intensive, and sample-dependent, as well as having a limited quantification capability in the case of confocal [144,148,226,227]. A standardized and quantified assay is needed to improve understanding of nanoparticle subcellular trafficking [226,228]. Quantification of subcellular localization has been demonstrated using raman spectroscopy. In raman spectroscopy, a determined fingerprint of the nanoparticle and associated organelle is determined and then points thorughout a raman map of a cell are analysed to determine the presence of the particle in a particular subcellular region. While this method works well for large organelles, the pixel size of a raman map is ~1 μm, limiting the resolution of smaller organelles such as endosomes [229,230]. The added advantage of raman spectroscopy, as well as the emergence of image correlation spectroscopy, is the ability to probe the local environment. In an interesting study, Gooding, Gaus and coworkers were able to moniter the delivery of nanoparticles into the nucleus and monitor the release of loaded doxorubicin from the nuclear localized nanoparticles using the pair correlation function (pCF) to identify barriers to diffusion [205]. Importantly, mechanistic insight can also be through the implementation of negative controls preventing subcellular trafficking and would also enhance the confidence of results [212].

Delivery to subcellular organelles is not extensively investigated, especially for polymeric nanoparticles, as it remains quite challenging to achieve robustly [144]. In 2015, a meta-analysis by Maity and Stepensky revealed that only 77 papers have investigated the active subcellular targeting of therapeutics using nanoparticles, with polymeric particles occupying just a fraction of these studies [148]. Compounding this, the majority of studies investigating strategies to improve subcellular trafficking involve the incubation of the nanoparticle with a cell. As a result, the ability to be internalized and escape from the endosome will also influence subcellular trafficking. Further research into how the nanoparticle structure can influence subcellular trafficking is an exciting opportunity for growth in the field of nanomedicine. 

## 6. Conclusions

Given the complex and interconnected makeup of biological barriers and their intrinsic nature to exclude exogenous material, it is understandable that numerous literature reports give dire accounts of the current state of nanomedicine. However, as highlighted by this review, there are numerous innovations in both the study of these barriers and the development of the tools required to overcome them. From novel imaging approaches through to exciting advances in material design, polymeric materials in particular highlight the diverse and interdisciplinary nature of the field. Polymeric constructs offer unique opportunities to understand mechanism and mode of action of therapeutics, combining the various advantages of modular design as described in this review. 

While not specifically addressed in this review, there is also increasing interest in developing strategies that “work with” biology, or potentially utilize administration routes that totally bypass one or more of the barriers discussed in this review. For example, in tumor immunotherapy it is becoming increasingly common to see direct injection of therapeutics intra-tumorally, rather than relying on systemic administration. This administration route could overcome some of the issues surrounding poor accumulation of intravenously administered nanomedicines due to unfavorable pharmacokinetics or biodistribution. Likewise, biology-modifying drugs can also be used to modulate the various barriers described in this review to enhance a particular response; one example discussed in this review was the use of vascular disrupting agents.

Irrespective of the strategies that are employed by nanotechnologists to develop novel polymeric nanomedicines, it will be through building solid foundational underpinnings of biological barriers and improving our knowledge of the interactions that occur at the bio-nano interface, where true breakthroughs will lie. Ultimately, this will lead to a more biologically-informed design of polymeric nanomedicines that is rationalized by clinical translation. As our ability to engineer polymers to overcome unfavorable interactions within biological systems progresses, the future of clinically-applicable personalized nanomedicines becomes increasingly optimistic. 

## Figures and Tables

**Table 1:** Tools to overcome the biological barriers of nanomedicine.

Biological Barrier	Tools to Overcome	Current Challenges	Referred Section
Interactions in the Blood Stream	Tuning Nanoparticle Physicochemical Properties Pre-Incubation/First pass Artificial Hard Corona	Protein corona has not been extensively profiled for soft nanoparticles The plasma protein content between patients varies	Section 2.1.1
			Section 2.1.2
Biodistribution	Nanoparticle Biophysical Properties Incorporation of Targeting Ligands Pre-Targeting Method Hitchhike onto Red Blood Cell	Defined sized cutoff for soft nanoparticle clearance remains challenging	Section 3.1 and Section 3.2
		Stealth property will be voided	Section 3.2
			Section 2.2
Gathering at Tumor Site	Nanoparticle Size Nanoparticle Hardness Vascular Normalization and Remodeling		Section 4.1.2
		Potential to induce metastasis	Section 4.1.3
Tumor Tissue Distribution	Nanoparticle Physicochemical Properties Charge-Switching Nanoparticles Size-Switching Nanoparticles Sheddable PEG Corona Macrophage Shuttling	Few studies report on the intratumoral distribution	Section 4.2.4
		Prevents use of environmentally-responsive polymers	
Receptor Affinity		Influenced by degree of opsonization	Section 4.2.3
Internalization	Nanoparticle Physicochemical Properties	Factors influencing nanoparticle internalization are not extensively investigated	Section 5.1.1
	Polymer Composition (e.g., Fluorous Substitution) Detachable Particle Corona Charge-Switching Particle Nanoparticle–Cell Membrane Fusion		
Endosomal Escape	pH-Responsive Materials that Membrane Interact or Swell Modifying the Therapeutic with Cell-Penetrating Peptides Modifying the Therapeutic Physicochemical Properties Incorporation of a Photosensitizer	Internalization must be faster than material activation at tumor microenvironment pH	Section 5.2.1
		Requires encapsulation for selective delivery	
		Limitations with depth of penetration of light and toxicity	
Subcellular Trafficking	(Nucleus) <9 nm Diameter of Nanoparticle or Therapeutic Conjugation of a Nuclear Localization Signal (Mitochondria) Incorporation of Mitochondrotropic Polymers Conjugation of a Mitochondria Targeting Signal	Factors influencing nanoparticle subcellular trafficking are not extensively investigated	Section 5.3.1
			Section 5.3.2

## Supplementary Files

Supplementary File 1
