# Supplementary material for: Engineered Polymeric Materials for Biological Applications: Overcoming Challenges of the Bio–Nano Interface
Source: Polymers (Basel). 2019 Sep 2;11(9):1441. doi: 10.3390/polym11091441 (PMC6780590; doi:10.3390/polym11091441)
Supplement: Supplementary File 1 [file polymers-11-01441-s001.zip › Escape Figure Permission/Escape figure citation/Bale_et_al-2010-Biotechnology_and_Bioengineering.pdf]

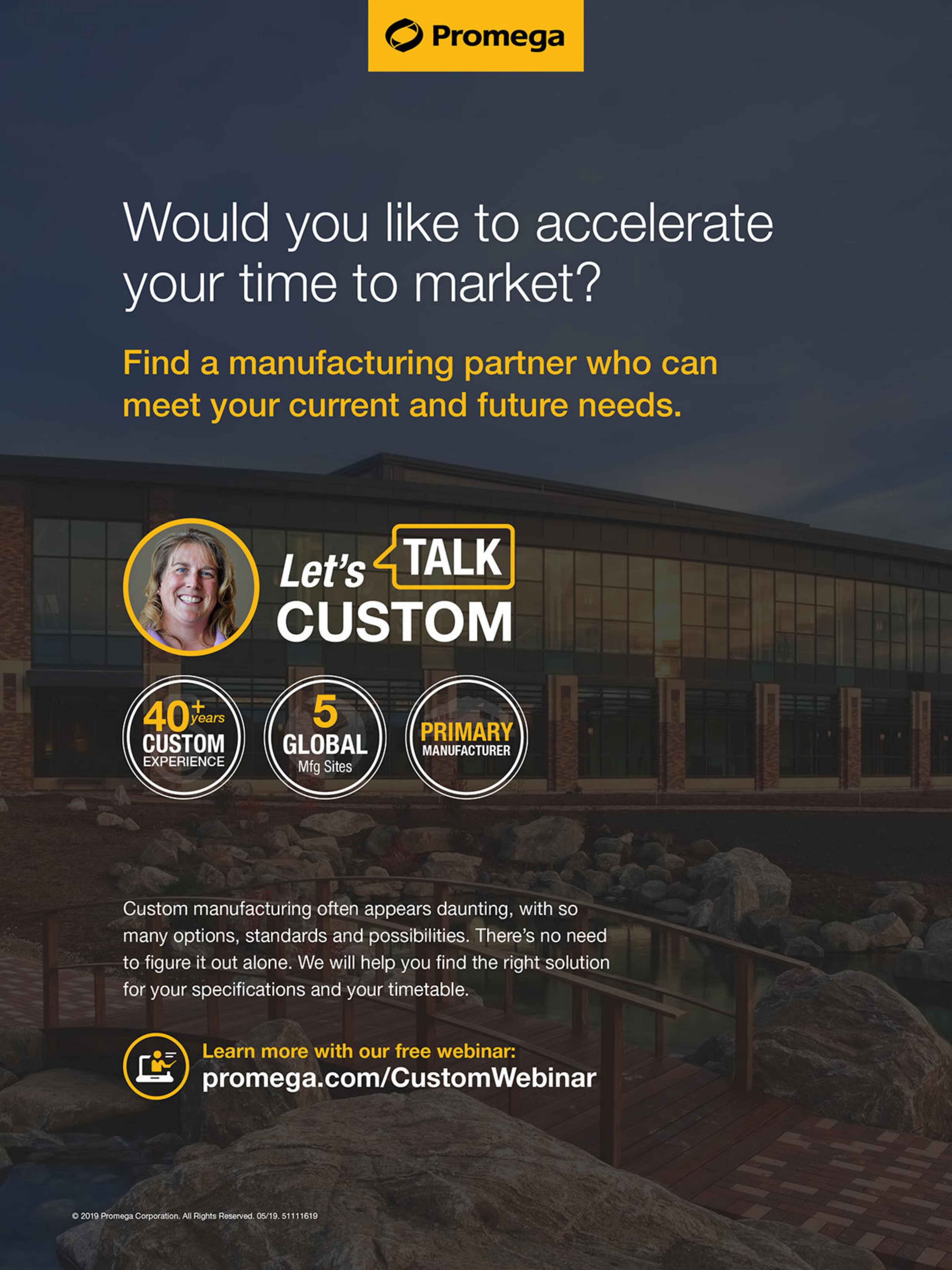
Promega
Would you like to accelerate your time to market?
Find a manufacturing partner who can meet your current and future needs.
Let's TALK CUSTOM
40+ years CUSTOM EXPERIENCE
5 GLOBAL Mfg Sites
PRIMARY MANUFACTURER
Custom manufacturing often appears daunting, with so many options, standards and possibilities. There's no need to figure it out alone. We will help you find the right solution for your specifications and your timetable.
Learn more with our free webinar:
promega.com/CustomWebinar
© 2019 Promega Corporation. All Rights Reserved. 05/19. 51111619

Communication to the Editor

Biotechnology and Bioengineering

A GFP Complementation System for Monitoring and Directing Nanomaterial Mediated Protein Delivery to Human Cellular Organelles

Shyam Sundhar Bale,[1,2] Seok Joon Kwon,[1,2] Dhiral A. Shah,[1] Ravi S. Kane,[1,2] Jonathan S. Dordick[1,2,3,4]

[1]Department of Chemical and Biological Engineering, Rensselaer Polytechnic Institute, Troy, New York 12180; telephone: 518-276-2899 (J.S.D.), 518-276-2536 (R.S.K.); fax: 518-276-2207; e-mail: dordick@rpi.edu; kaner@rpi.edu

[2]Center for Biotechnology and Interdisciplinary Studies, Rensselaer Polytechnic Institute, Troy, New York 12180

[3]Department of Biomedical Engineering, Rensselaer Polytechnic Institute, Troy, New York 12180

[4]Department of Biology, Rensselaer Polytechnic Institute, Troy, New York 12180

Received 13 June 2010; revision received 18 July 2010; accepted 23 July 2010

Published online 17 August 2010 in Wiley Online Library (wileyonlinelibrary.com). DOI 10.1002/bit.22897

ABSTRACT: Protein-based therapeutics are gaining importance for their biocompatibility and activity toward specific targets. When these targets are intracellular, it is critical to deliver biomolecules to sites in the cell cytoplasm while retaining biomolecule activity in the complex cellular milieu. However, intracellular protein delivery is not viable unless accompanied by an active uptake mechanism or carrier mediated delivery. Moreover, once entry into the cell is achieved, detection of the biomolecule requires laborious techniques that lack real-time measurement. We have developed a fluorescence-based complementary protein delivery sensing system using split green fluorescence protein (GFP_{1-10} and GFP_{11}) fragments, which can be used as an indicator for protein delivery and retention of activity, and as a means to pinpoint subcellular localization. We demonstrate in vitro localized delivery by expressing the GFP_{11} fragment onto the mitochondrial outer membrane of human cells, and using a model carrier (15 nm silica nanoparticles) to deliver GFP_{1-10} and image trafficking and mitochondrial localization of protein delivery. Our results indicate that nanoscale materials can be used as protein carriers for targeting cell constituents including functional molecules, signaling pathways, and organelles. We envision that this GFP complementation system is ideally suited for directing nanoparticle-based delivery of drugs and other bioactive molecules into subcellular locations within cells, which can impact protein–protein interactions, signal transduction pathways, and organelle function in vitro within the context of high-throughput screening protocols.

Biotechnol. Bioeng. 2010;107: 1040–1047.

© 2010 Wiley Periodicals, Inc.

KEYWORDS: GFP complementation; nanoparticle–protein delivery; sub-cellular localization; protein delivery sensor; mitochondrial delivery

Protein delivery is a powerful tool to alter and influence cellular components and biological functions by mediating protein–protein interactions, protein interference in cell signaling pathways and intracellular trafficking (Leader et al., 2008). Targets for protein delivery can include receptors on the cell surface, proteins and signaling molecules in the cell cytosol, and cell organelles (Gibbs, 2000; Leader et al., 2008; Reichert and Valge-Archer, 2007; Schrama et al., 2006; Vescovi et al., 2006). While the vast majority of protein delivery routes have targeted cell surface receptors, the ability to target specific biomolecules, biomolecular assemblies, and organelles within cells provides a tantalizing array of possibilities to control cell function and fate, yet poses a host of potential problems (Boddapati et al., 2008; Savic et al., 2003). Two of the more important problems are retention of delivered protein activity and stability in the potentially harsh cellular milieu (e.g., the presence of proteases, change in pH), and efficient delivery of the protein without compartmentalization and degradation (Futami et al., 2005; Jiang et al., 2008; Kam et al., 2004; Kostarelos et al., 2007; Lee et al., 1996; Provoda et al., 2003; Slowing et al., 2007a,b; Torchilin, 2006). Furthermore, site selective delivery to sub-cellular components is crucial for targeting selectively individual cell components. The use of nanoparticles may address these concerns. For example, in our previous work, we showed that silica nanoparticles (15 ± 5 nm) functionalized with

Shyam Sundhar Bale and Seok Joon Kwon contributed equally to this work.
Correspondence to: J.S. Dordick or R.S. Kane
Contract grant sponsor: National Science Foundation (NSF-NSEC)
Contract grant number: DMR-0642573
Additional Supporting Information may be found in the online version of this article.

© 2010 Wiley Periodicals, Inc.

n-octadecyltrimethoxysilane (SiNP-ODMS) were rapidly internalized by mammalian cells, resulting in the efficient delivery of proteins that could alter cell fate (Bale et al., 2010).

In the current work, we have exploited protein complementation of green fluorescence protein (GFP) to address both the sensing of nanoparticle–protein conjugate cellular delivery and directing the delivered nanoparticles–protein conjugates to specific locations within the cell. With respect to the former, visualization of protein delivery into cells traditionally has required fluorescent tagging of the protein or immunofluorescence assays. Tagging, however, requires a chemically modified protein, which may affect biomolecular activity and stability, while immunofluorescence generally requires cell fixation and permeabilization, which is laborious and prohibits real-time analysis of live cells. By employing fragments of GFP, specifically GFP_{1-10} and GFP_{11}, we demonstrate that it is possible to visualize in real time nanoparticle–protein conjugate delivery, as well as to target site specific locations within the cell (Cabantous and Waldo, 2006; Cabantous et al., 2005). A split-GFP system has also been shown to identify cytosolic localization of proteins in mammalian cells (Van Engelenburg and Palmer, 2010). In this case, a bacterial secretion system was used to introduce proteins into host cells.

Our approach is highlighted in Scheme 1 and facilitated by the well-known complementation of split GFP fragments (Cabantous and Waldo, 2006; Cabantous et al., 2005; Huang and Bystroff, 2009). GFP can be prepared in two fragments by removing a 15 amino acid segment (comprising strand 11), thereby resulting in a large GFP_{1-10} (214 amino acids) and the short GFP_{11} peptide. When the two fragments are brought together, complementation occurs and the protein's chromophore is allowed to form, resulting in the distinct GFP fluorescence. Importantly, in the absence of strand 11, and prior to chromophore maturation, GFP_{1-10} has minimal fluorescence. Thus, it occurred to us that delivery of GFP_{1-10} on SiNP-ODMS into a cell cytosol that contains the GFP_{11} peptide would result in complementation and formation of green fluorescence (Scheme 1). Furthermore, if the GFP_{11} fragment were genetically fused to proteins present at selected locations within the cell, that is, an organelle, this complementation system would yield highly localized fluorescence at the site of complementation.

To enable our approach, we initially sought to assess whether our GFP complementation system was functional in an in vitro, cell-free system, which would also provide information on the kinetics of chromophore maturation. The fragments were obtained by transfecting *Escherichia coli (E.Coli)* cells with two plasmids, pET-GFP_{1-10} and pCDF-intein-GFP_{11} to produce the protein fragments GFP_{1-10} and GFP_{11}, respectively (Huang and Bystroff, 2009), which were purified using Ni-NTA column chromatography. GFP_{11} is expressed as intein-GFP_{11}, which enables stable expression of GFP_{11} as a fusion protein—the small size of the GFP_{11} prevents stable expression on its own—and the self-splicing activity of the intein allows rapid liberation of the GFP_{11} protein fragment into the cell cytosol. The purified fragments were spotted on a microscope slide and the image scanned using a microarray scanner (Figs. 1a and S1). A clear increase in fluorescence was evident when both protein fragments were present together, whereas only minimal background fluorescence was observed from either of the fragments when incubated alone, which indicates that complementation was successful. Native GFP requires up to 4 h once folded to form the mature chromophore (Reid and

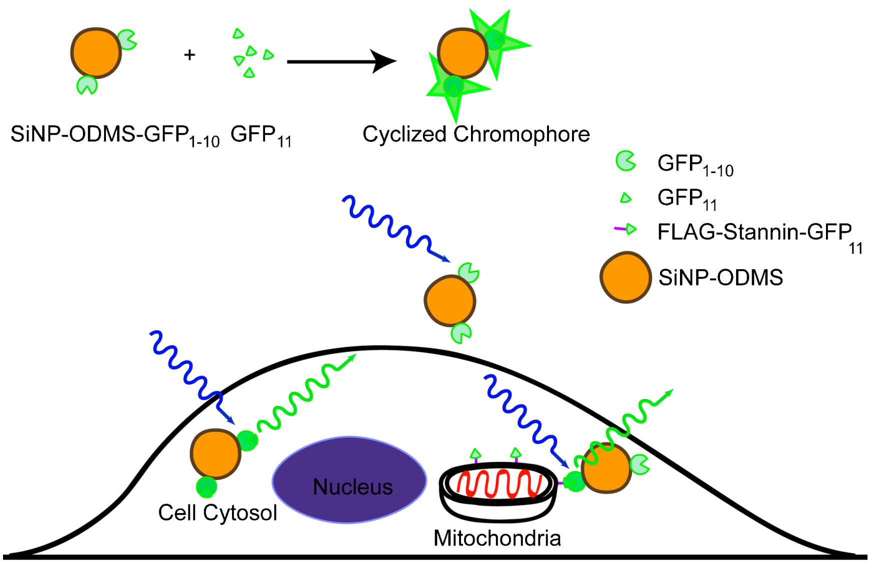

Scheme 1. Strategy of GFP complementation to sense protein delivery. The larger protein fragment GFP_{1-10} is immobilized and the indicator peptide GFP_{11} is expressed: (**a**) in the cell cytosol; or (**b**) on mitochondria. A cyclized (mature) chromophore is formed only when the nanoparticle–protein conjugates complement at target locations. [Color figure can be seen in the online version of this article, available at wileyonlinelibrary.com.]

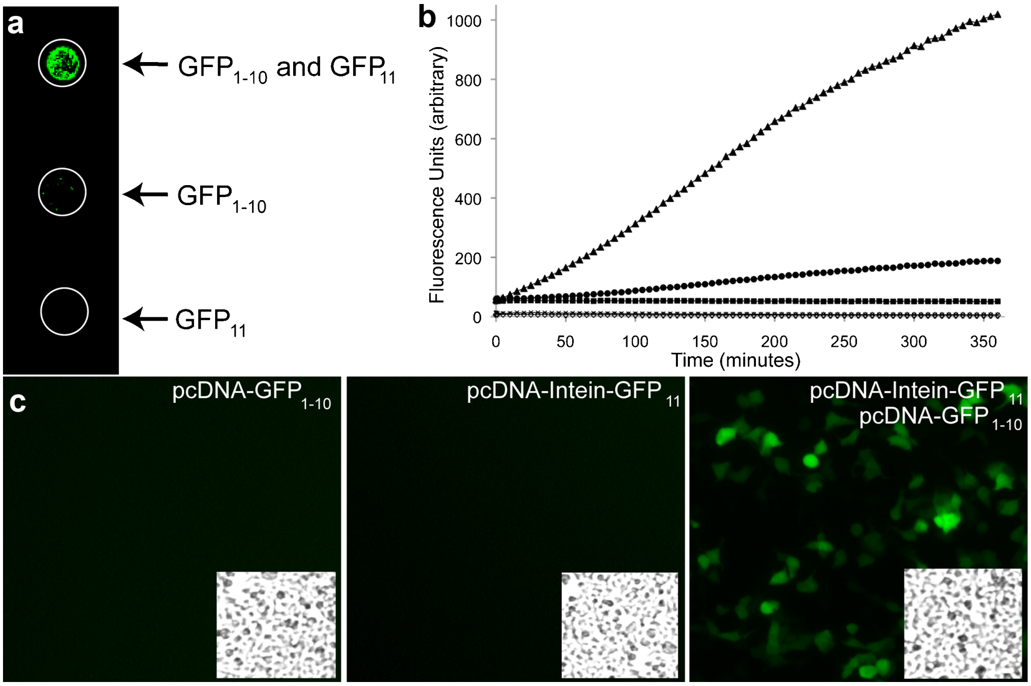

Figure 1. **a**: In vitro complementation of GFP_{1-10} and GFP_{11} fragments (expressed in *E. coli*). **b**: Kinetics of GFP complementation. Fluorescence profiles of GFP_{1-10} (○), GFP_{11} (■), GFP_{1-10} and GFP_{11} (▲), SiNP-ODMS-GFP_{1-10} (×), and SiNP-ODMS-GFP_{1-10} and GFP_{11} (●). **c**: Cell-based in vitro complementation of GFP_{1-10} and GFP_{11} fragments in cell cytosol of HEK-293 cells. [Color figure can be seen in the online version of this article, available at wileyonlinelibrary.com.]

Flynn, 1997; Ward and Bokman, 1982; Yang et al., 1996). As shown in Figure 1b, the complementation system required essentially the same amount of time to yield maximal fluorescence, which is consistent with reports in the literature (Cabantous et al., 2005; Huang and Bystroff, 2009).

We next proceeded to evaluate whether complementation could occur in an in vitro cell-based system by constructing two mammalian expression vectors, pcDNA-GFP_{1-10} and pcDNA-intein-GFP_{11} for the expression of GFP_{1-10} and GFP_{11} fragments, respectively, in human cells. The human embryonic kidney (HEK-293) cell line served as an excellent model host for in vitro cell-based complementation due to its ease of transfection using the Lipofectamine Plus reagent (Invitrogen, CA, USA). HEK-293 cells were transfected with either pcDNA-GFP_{1-10} or pcDNA-intein-GFP_{11}, or both together. Successful cell-based in vitro complementation was achieved only by the co-expression of GFP_{1-10} and the intein-GFP_{11} fragment (which produces the GFP_{11} peptide after intein-mediated protein splicing) in the cytosol (Fig. 1c). Thus, cell-based in vitro complementation could be achieved, and in particular, the intein-GFP_{11} expression system provided an excellent intracellular "label" for use in localized protein delivery.

Having demonstrated that co-expression of GFP_{1-10} and GFP_{11} could lead to complementation, we focused our attention on determining whether complementation could occur if one of the two fragments was added exogenously to the cell using the SiNP-ODMS delivery system. For these experiments, we chose MCF-7 (human breast cancer cells) cells, which were shown in our previous work to actively internalize nanoparticle–protein conjugates and affect cell function by targeting cellular signaling pathways (Bale et al., 2010). To this end, we attached GFP_{1-10} to nanoparticles using our previously established procedure (Bale et al., 2010) and incubated 0.1 mg/mL of SiNP-ODMS-GFP_{1-10} conjugates with MCF-7 cells transfected with pcDNA-intein-GFP_{11}. Figure 2 shows the confocal microscopy images of cells at the end of 1, 4, and 14 h incubation. The cells were stained with membrane stain (DiI) prior to the addition of nanoparticles, and a cell nucleus stain (DAPI, dilactate) was added 10 min before imaging. The images reveal a steady increase in the intracellular fluorescence, indicative of succesful complementation in the cell cytosol with the GFP_{11} fragment expressed in the cell (Fig. 2). Moreover, the increase in fluorescence during this time is consistent with the maintenance of GFP_{1-10} cytosolic stability on SiNP-ODMS. Furthermore, the gain of fluorescence indicates that the nanoparticle–protein conjugates escaped from the vesicular compartments (endosomes/lysosomes) to the cytosol.

We further tested the cytotoxicity and uptake of these nanoparticle–protein conjugates using Trypan Blue assay and fluorescence assisted cell sorting (FACS). MCF-7 cells were incubated with increasing concentrations of SiNP-ODMS-GFP_{1-10} conjugates for 14 h followed by

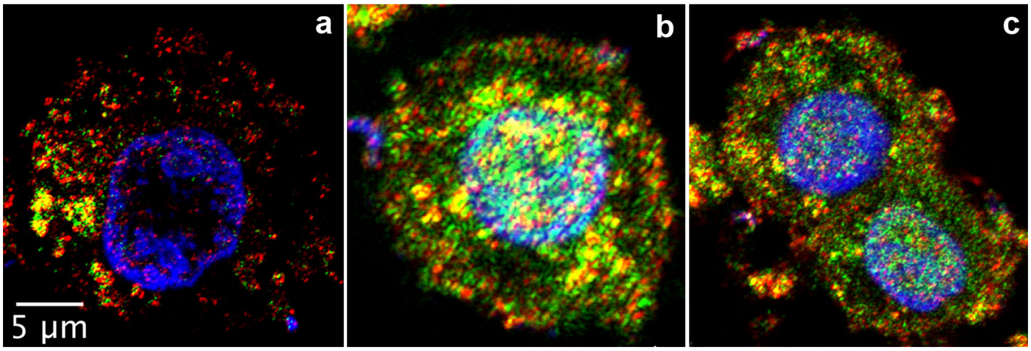

Figure 2. Confocal microscopy images showing uptake and complementation of SiNP-ODMS-GFP_{1-10} conjugates in MCF-7 cells after an incubation of (**a**) 1 h; (**b**) 4 h; and (**c**) 14 h. The cell membrane was stained with DiI prior to addition of SiNP-ODMS-GFP_{1-10} conjugates. [Color figure can be seen in the online version of this article, available at wileyonlinelibrary.com.]

measurement of cell viability with the Trypan Blue reagent (Fig. 3a). Cell viability was ca. 80% at concentrations of the conjugates as high as 0.8 mg/mL, indicating the absence of significant nanoparticle-derived toxicity for these nanoparticle–protein conjugates. To observe cellular uptake of the conjugates, we incubated 0.1 mg/mL of SiNP-ODMS-GFP_{1-10} conjugates with cells transfected with pcDNA-intein-GFP_{11} and analyzed the cells using FACS. As a control, we incubated 0.1 mg/mL of SiNP-ODMS-GFP_{1-10} conjugates with cells that did not express GFP_{11}, wherein no fluorescence was expected. As seen in Figure 3b, there was no change in the fluorescence of cells after incubation with SiNP-ODMS; however there was a strong shift of the histogram when cells expressing GFP_{11} were incubated with SiNP-ODMS-GFP_{1-10}. This information (Fig. 3c) indicates that nearly all the cells receive SiNP-protein conjugates, as reflected in an increase in the cytosolic fluorescence. As expected, in the absence of GFP_{11} expression, no fluorescence was observed (data not shown).

The retention of GFP structure and function post-delivery encouraged us to target cellular organelles. Experiments by Waldo et al. have shown the flexibility of using GFP_{11} as a tag to monitor protein folding (Cabantous et al., 2005). Given the specificity of complementation, we expected that such specificity could be used to target organelles, such as mitochondria. Owing to their role in a variety of cellular functions, such as energy production and control of apoptosis, mitochondria have been one of the most studied cell organelles for aberrant cell behavior (Boddapati et al., 2008; Fulda and Kroemer, 2009; Taylor and Turnbull, 2005; Tuppen et al., 2010; Zeviani and Di Donato, 2004). To this end, we engineered a plasmid to produce the protein fragment GFP_{11} fused to the C-terminus of the target protein, stannin (Billingsley et al., 2006; Buck-Koehntop et al., 2005; Davidson et al., 2004; Dejneka et al., 1997) that is expressed primarily on the outer membrane of mitochondria. Stannin is an 88 amino acid protein with a single trans-membrane helix, which is primarily found localized in mitochondria, although some minor localization is observed in other vesicular organelles. Furthermore, the C-terminal domain of stannin is cytoplasmic, which offers a favorable site for expressing GFP_{11} (Billingsley et al., 2006; Buck-Koehntop et al., 2005). Accordingly, we designed a plasmid pcDNA-FSL-GFP_{11}, using assembly PCR to express FLAG-

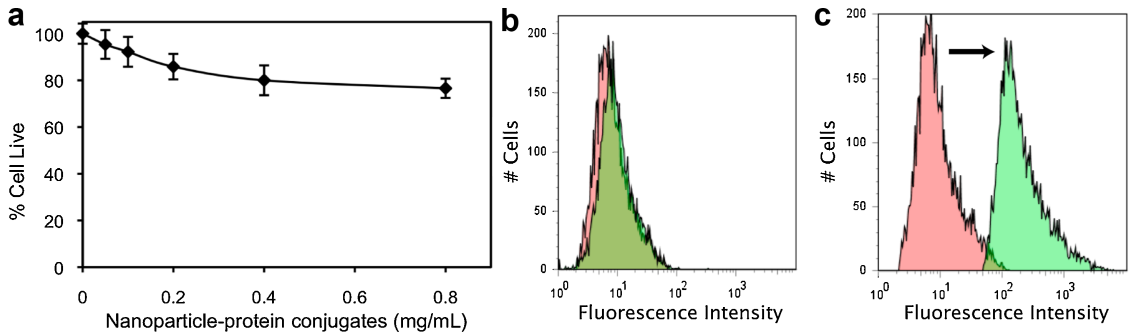

Figure 3. **a**: Dose response cytotoxicity profile of SiNP-ODMS-GFP_{1-10} conjugates. Trypan Blue assay results showing cell survival after an incubation of 14 h. **b**: FACS histograms comparing the FITC values for cells only (red) and cells incubated with SiNP-ODMS-GFP_{1-10} (green) that do not express GFP_{11}. **c**: FACS histograms comparing the FITC values for cells only (red) and cells incubated with SiNP-ODMS-GFP_{1-10} (green) with cells expressing GFP_{11}. [Color figure can be seen in the online version of this article, available at wileyonlinelibrary.com.]

stannin-GFP_{11} that would localize in the cytosol, specifically on the mitochondrial outer membrane. For these experiments, we chose HEK-293 cells for the ease of transfection when compared with MCF-7 cells. To test cell-based in vitro complementation with the fusion protein, we transfected HEK-293 cells with pcDNA-FSL-GFP_{11} and pcDNA-$GFP_{1–10}$. The successful complementation of both fragments was observed as localization of fluorescence on mitochondria, which were independently stained using mitotracker (Invitrogen, CA, USA). As seen in Figures 4a, S2, and S3, successful complementation was indicated by the favorable expression of the GFP_{11} fragment on the mitochondria. The majority of expression occurred on the mitochondria, thus demonstrating efficient localization.

To monitor in vitro protein delivery to mitochondria, $GFP_{1–10}$ protein was purified from an *E. coli* culture and attached to dye-doped silica nanoparticles (Life Sciences Inc, FL, USA) Rubpy doped nanoparticles enabled us to track the nanoparticles independent of the fluorescence from the complemented GFP in the cells. We proceeded to transfect HEK-293 cells with pcDNA-FSL-GFP_{11} to express stannin-GFP_{11} in the cell cytosol and localized on the mitochondrial outer membrane and then incubated the cells with SiNP-ODMS-$GFP_{1–10}$ conjugates for 24 h. To identify localization within the cells, mitochondria were stained using mitotracker and observed by confocal microscopy, which showed clear uptake of the conjugates and localization of the conjugates to the mitochondrial outer membrane (Figs. 4b and S4). Line scans show the localization of nanoparticle–protein conjugates as seen by the merge with mitotracker, which is realized only by the successful complementation with stannin-GFP_{11} expressed on the mitochondria. The $GFP_{1–10}$ remained bound to SiNP-ODMS and did not desorb from the nanoparticles; such a result may be important for nanoparticles to be used ultimately as carriers for protein immobilization, delivery, and retention of function of immobilized biomolecules in the cellular milieu. Control experiments with cells not transfected with pcDNA-FSL-GFP_{11} and incubated with SiNP-ODMS-$GFP_{1–10}$ showed no fluorescence (Fig. S5) indicating that GFP complementation was highly selective.

In conclusion, we have demonstrated a complementary sensing system for protein delivery, which may be applicable to selective target-based screening of therapeutic protein molecules, and efficient biomolecular delivery into cells, sensing protein–protein interactions and imaging protein delivery. Moreover, this complementation approach may be useful to direct nanoparticle-based drug/bioactive molecule carriers to specific compartments within a cell, which can be used to produce loss or gain of function in vitro. This may thus serve as a useful tool in high-throughput screening.

Materials and Methods

Gene Cloning

The genes encoding $GFP_{1–10}$ and fusion protein of intein and GFP_{11} were synthesized by assembly PCR protocol

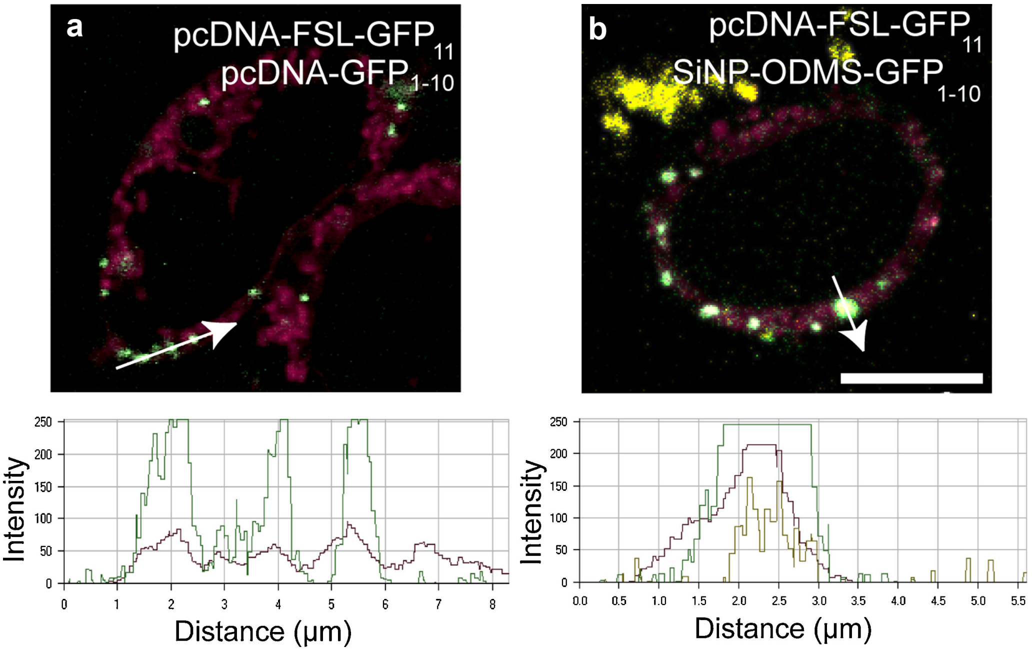

Figure 4. **a**: Confocal microscopy images showing cell-based in vitro complementation of $GFP_{1–10}$ and stannin-GFP_{11} fragments in HEK-293 cells. Line scans show the fluorescence profiles of complemented GFP (green) and mitotracker (pink). **b**: Confocal microscopy showing uptake and complementation of SiNP-ODMS-$GFP_{1–10}$ conjugates with stannin-GFP_{11} in HEK-293 cells. Line scans show the fluorescence profiles of nanoparticles (yellow), complemented GFP (green) and mitotracker (pink). Scale bar is 10 μm. [Color figure can be seen in the online version of this article, available at wileyonlinelibrary.com.]

(Hoover and Lubkowski, 2002) and cloned in pET28a (Novagen, Darmstadt, Germany) and pCDF-1b (Novagen), to construct pET-GFP_{1-10} and pCDF-intein-GFP_{11}, respectively. GFP_{1-10} and intein-GFP_{11} were PCR amplified and subcloned in pcDNA 3.3-TOPO (Invitrogen) to construct pcDNA-GFP_{1-10} and pcDNA-intein-GFP_{11}, respectively, for expression in human cells. To construct the gene encoding the fusion protein of FLAG-stannin-GFP_{11}, we amplified the gene encoding FLAG-stannin (Accession number: AF030196) from human cDNA library (Stratagene, La Jolla, CA, USA) by PCR using two primers: the forward primer 5′-ATGGATT ACAAGGATGACGACGATAAGATGTCTATTATGGACC-ACAGCC-3′, and the reverse primer 5′-TGATCCTGAT CCGCCACCGCCGTGGACTTCCGG-3′. Simultaneously, the gene encoding GFP_{11} was amplified by PCR using two primers: the forward primer 5′-GGTGGCGGATCAG GATCAAAGAGAGACCACATGGTCC-3′, and the reverse primer 5′-TTATCATGTAATCCCAGCAGC-3′. Two PCR products were reassembled by PCR using two primers: the forward primer 5′-ATGGATTAC AAGGATGACGAC-3′, and the reverse primer 5′-TTATCATGTAATCCCAGCA GC-3′. The PCR products encoding FLAG-stannin-GFP_{11} were subcloned in pcDNA 3.3-TOPO (Invitrogen) to construct pcDNA-FSL-GFP_{11}.

Protein Expression and Purification From *E. coli*

N-terminal His_6-tagged GFP_{1-10} was expressed in BL21 (DE3) *E. coli* cells. For obtaining GFP_{1-10}, *E. coli* cells transfected with the plasmid pET-GFP_{1-10} were incubated in a 5 mL LB-media culture with 1% ampicillin, overnight at 37°C and 200 rpm to obtain the seed culture. This was then transferred into a larger, 500 mL culture with 1% ampicillin and grown at 37°C at 200 rpm for 3–4 h (OD = 0.4–0.6). Protein expression was induced by adding 1% isopropyl β-D-1-thiogalactopyranoside to the culture and shaken at room temperature at 200 rpm overnight. Cells were harvested and lysed using lysozyme and freeze–thaw cycles. Histidine-tagged GFP_{1-10} was purified using a Ni-NTA column (Invitrogen) using the manufacturer's protocol.

Mammalian Cell Culture

Human embryonic kidney (HEK-293) cells, breast cancer (MCF-7) cells (American Type Cell Culture Collection, Manassas, VA, USA) were maintained in Dulbecco's modified eagle's medium (DMEM) supplemented with 5% (v/v) fetal bovine serum (FBS) and 1% (v/v) penicillin–streptomycin mixture. Cell cultures were maintained at 37°C and in 5% CO_2. For transfecting plasmids, cells were seeded in a 12-well plate at 3×10^5 cells/well and allowed to reach 70% confluency. Plasmids were transfected using Lipofectamine Plus reagent (Invitrogen) using the standard protocol provided. Briefly, 500 ng of plasmid was incubated with 1 μL of Plus Reagent for 5 min. To this mixture, 3 μL of Lipofectamine reagent diluted in 200 μL of serum-free OPTI-MEM media was added and incubated for 30 min. DMEM media was replaced with the plamsid mixture in serum free OPTI-MEM media and incubated for at least 3 h. At the end of incubation media was replaced with DMEM with 5% FBS.

Functionalization of Nanoparticles

Silica nanoparticles were first functionalized with *n*-octadecyltrimethoxysilane (*n*-ODMS). Briefly, ca. 8 mg of silica nanoparticles (diameter of 15 ± 5 nm, EKA Chemicals, Inc., Augusta, GA) were added to 1 mL dry ethanol and washed at least three times by centrifugation at 10,000 rpm for 5 min followed by re-dispersion in dry ethanol by sonication. Washed nanoparticles were then sonicated in a solution of 1% (v/v) *n*-ODMS in dry ethanol for 2 h. *n*-ODMS functionalized silica nanoparticles were washed in dry ethanol three times followed by three washes in sterile phosphate buffer saline (PBS) by repeated centrifugation and sonication as described above.

To attach proteins, *n*-ODMS functionalized nanoparticles were suspended in sterile PBS and a solution of protein in PBS at a concentration of 2 mg/mL and shaken at 200 rpm for 2 h at 4°C. The resulting nanoparticle–protein conjugates were washed three times in PBS by repeated centrifugation at 7,000 rpm for 5 min and re-dispersion by pipetting. Supernatants from all of the washes were collected and the protein content was analyzed using the bicinchoninic acid assay (Pierce Biotechnology, Rockford, IL). SiNP-protein conjugates were used immediately or stored at 4°C.

Confocal Microscopy

Glass bottom petri dishes (35 mm total diameter, 22 mm glass bottom; Electron Microscopy Sciences, PA, USA) were coated with poly-L-ornithine (0.01 mg/mL in sterile PBS) by overnight incubation and stored at −20°C. Poly-L-ornithine coated petri dishes were washed with sterile PBS three times before use. For confocal microscopy, approximately 1×10^5 cells were incubated in a glass bottom petri dish coated with poly-L-ornithine. Cells were allowed to settle for 15 min and 1 mL of fresh media was added to the wells and incubated. Cells were washed with media prior to addition of nanoparticle–protein conjugates suspended in 1 mL of fresh media. For plamsid transfection, plated cells were transfected using the Lipofectamine Plus reagent (Invitrogen, CA, USA).

Staining Protocol

The position and integrity of the internalized nanoparticle–protein conjugates were evaluated by confocal microscopy. For these experiments, we stained the nucleus, mitochondria, and cell membrane compartments of the cell. The

stains were added to the cell media and mixed. The nucleus and mitochondria were stained using DAPI (dilactate) and mitotracker, respectively. Ten microliters of DAPI ($\lambda_{ex} = 405$ nm, $\lambda_{em} = 460$ nm, 0.1 μM), 10 μL of mitotracker ($\lambda_{ex} = 633$ nm, $\lambda_{em} = 650$ nm, 10 μM) were added and incubated for 10 min. Cell membranes were stained using 10 μL of DiI ($\lambda_{ex} = 553$ nm, $\lambda_{em} = 570$ nm, 0.1 μM), which was added to the cells along with the nanoparticles. For tracking nanoparticle–protein conjugates, dye doped silica nanoparticles containing Rubpy ($\lambda_{ex} = 458$ nm, $\lambda_{em} = 592$ nm) were conjugated with fragment of GFP_{1-10}. Complemented GFP ($GFP_{1-10} + GFP_{11}$, GFP_{1-10} + stannin-GFP_{11}) was observed using $\lambda_{ex} = 488$ nm, $\lambda_{em} = 515$ nm. Stained samples were washed thoroughly with 1 mL of fresh DMEM at least three times and imaged.

Toxicity Assay

Cells were seeded in a 12-well plate at a density of 3×10^5 cells/well and incubated for 4–6 h. Cells were then washed with DMEM and incubated overnight with a solution of nanoparticle–protein conjugates diluted in fresh media. Cell media from each sample was collected and cells were detached by incubating with 300 μL of 0.05% Trypsin–EDTA mixture. Detached cells were then suspended in the media previously collected from the sample. Ten microliters of Trypan Blue was added to 10 μL of the cell suspension and live and dead cells were counted on a hemocytometer.

Fluorescence Assisted Cell Sorting

Cells were seeded in a 12-well plate at a density of 3×10^5 cells/well and incubated for 4–6 h. The cells were then washed with DMEM and incubated overnight with a solution of nanoparticle–protein conjugates diluted in fresh media. For FACS analysis, cells were washed with fresh DMEM, PBS, and then detached from the surface by incubating with 300 μL of 0.05% Trypsin–EDTA mixture. Samples collected were resuspended in DMEM. Nanoparticle–protein conjugates not internalized into the cells were removed from the cell suspension by centrifuging the cells at 1,000 rpm for 3 min and resuspending cells in fresh DMEM. The resulting cell suspension was diluted in an equal volume of FACS Flow (BD Biosciences, San Jose, CA, USA) followed by analysis on FACS.

The cell suspensions were analyzed on a BD Sciences LSRII Flow Cytometer. The forward scatter and side scatter for the cells were adjusted using MCF-7 cells (control) to a range of 50–100, and were appropriately gated to include the majority of live cell population. The Fluorescein Isothiocyanate (FITC)-A signal ($\lambda_{ex} = 488$ nm, $\lambda_{em} = 515$ nm) 515 nm) for cells without fluorophore was adjusted to obtain the background signal for control cells. Cells suspensions with nanoparticle-conjugates were analyzed with the same settings and at least 5,000 events were obtained for each sample, in the gate selected for control cells. Collected data were analyzed and plotted using Flow Jo.

This work was supported by the National Science Foundation through NSF-NSEC grant DMR-0642573. We thank Prof. C. Bystroff for the kind donation of plasmids pET-GFP_{1-10} and pCDF-intein-GFP_{11}.

References

Bale SS, Kwon SJ, Shah DA, Banerjee A, Dordick JS, Kane RS. 2010. Nanoparticle-mediated cytoplasmic delivery of proteins to target cellular machinery. Acs Nano 4(3):1493–1500.

Billingsley ML, Yun J, Reese BE, Davidson CE, Buck-Koehntop BA, Veglia G. 2006. Functional and structural properties of stannin: Roles in cellular growth, selective toxicity, and mitochondrial responses to injury. J Cell Biochem 98(2):243–250.

Boddapati SV, D'Souza GGM, Erdogan S, Torchilin VP, Weissig V. 2008. Organelle-targeted nanocarriers: Specific delivery of liposomal ceramide to mitochondria enhances its cytotoxicity in vitro and in vivo. Nano Lett 8(8):2559–2563.

Buck-Koehntop BA, Mascioni A, Buffy JJ, Veglia G. 2005. Structure, dynamics, and membrane topology of stannin: A mediator of neuronal cell apoptosis induced by trimethyltin chloride. J Mol Biol 354(3):652–665.

Cabantous S, Waldo GS. 2006. In vivo and in vitro protein solubility assays using split GFP. Nat Method 3(10):845–854.

Cabantous S, Terwilliger TC, Waldo GS. 2005. Protein tagging and detection with engineered self-assembling fragments of green fluorescent protein. Nat Biotechnol 23(1):102–107.

Davidson CE, Reese BE, Billingsley ML, Yun JK. 2004. Stannin, a protein that localizes to the mitochondria and sensitizes NIH-3T3 cells to trimethyltin and dimethyltin toxicity. Mol Pharmacol 66(4):855–863.

Dejneka NS, Patanow CM, Polavarapu R, Toggas SM, Krady JK, Billingsley ML. 1997. Localization and characterization of stannin: Relationship to cellular sensitivity to organotin compounds. Neurochem Int 31(6):801–815.

Fulda S, Kroemer G. 2009. Targeting mitochondrial apoptosis by betulinic acid in human cancers. Drug Discov Today 14(17–18):885–890.

Futami J, Kitazoe M, Maeda T, Nukui E, Sakaguchi M, Kosaka J, Miyazaki M, Kosaka M, Tada H, Seno M, Sasaki J, Huh N-H, Namba M, Yamada H. 2005. Intracellular delivery of proteins into mammalian living cells by polyethylenimine-cationization. J Biosci Bioeng 99(2):95–103.

Gibbs JB. 2000. Mechanism-based target identification and drug discovery in cancer research. Science 287(5460):1969–1973.

Hoover DM, Lubkowski J. 2002. DNAWorks: An automated method for designing oligonucleotides for PCR-based gene synthesis. Nucleic Acids Res 30(10):e43.

Huang YM, Bystroff C. 2009. Complementation and reconstitution of fluorescence from circularly permuted and truncated greed fluorescent protein. Biochemistry 48(5):929–940.

Jiang W, Kim BYS, Rutka JT, Chan WCW. 2008. Nanoparticle-mediated cellular response is size-dependent. Nat Nanotechnol 3(3):145–150.

Kam NWS, Jessop TC, Wender PA, Dai HJ. 2004. Nanotube molecular transporters: Internalization of carbon nanotube-protein conjugates into mammalian cells. J Am Chem Soc 126(22):6850–6851.

Kostarelos K, Lacerda L, Pastorin G, Wu W, Wieckowski S, Luangsivilay J, Godefroy S, Pantarotto D, Briand JP, Muller S, Prato M, Bianco A. 2007. Cellular uptake of functionalized carbon nanotubes is independent of functional group and cell type. Nat Nanotechnol 2(2):108–113.

Leader B, Baca QJ, Golan DE. 2008. Protein therapeutics: A summary and pharmacological classification. Nat Rev Drug Discov 7(1):21–39.

Lee KD, Oh YK, Portnoy DA, Swanson JA. 1996. Delivery of macromolecules into cytosol using liposomes containing hemolysin from Listeria monocytogenes. J Biol Chem 271(13):7249–7252.

Provoda CJ, Stier EM, Lee KD. 2003. Tumor cell killing enabled by listeriolysin O-liposome-mediated delivery of the protein toxin gelonin. J Biol Chem 278(37):35102–35108.

Reichert JM, Valge-Archer VE. 2007. Development trends for monoclonal antibody cancer therapeutics. Nat Rev Drug Discov 6(5):349–356.

Reid BG, Flynn GC. 1997. Chromophore formation in green fluorescent protein. Biochemistry 36(22):6786–6791.

Savic R, Luo LB, Eisenberg A, Maysinger D. 2003. Micellar nanocontainers distribute to defined cytoplasmic organelles. Science 300(5619):615–618.

Schrama D, Reisfeld RA, Becker JC. 2006. Antibody targeted drugs as cancer therapeutics. Nat Rev Drug Discov 5(2):147–159.

Slowing II, Trewyn BG, Lin VSY. 2007a. Mesoporous silica nanoparticles for intracellular delivery of membrane-impermeable proteins. J Am Chem Soc 129(28):8845–8849.

Slowing II, Trewyn BG, Giri S, Lin VSY. 2007b. Mesoporous silica nanoparticles for drug delivery and biosensing applications. Adv Funct Mater 17(8):1225–1236.

Taylor RW, Turnbull DM. 2005. Mitochondrial DNA mutations in human disease. Nat Rev Genet 6(5):389–402.

Torchilin VP. 2006. Recent approaches to intracellular delivery of drugs and DNA and organelle targeting. Annu Rev Biomed Eng 8:343–375.

Tuppen HAL, Blakely EL, Turnbull DM, Taylor RW. 2010. Mitochondrial DNA mutations and human disease. Biochim Biophys Acta Bioenerg 1797(2):113–128.

Van Engelenburg SB, Palmer AE. 2010. Imaging type-III secretion reveals dynamics and spatial segregation of Salmonella effectors. Nat Method 7(4):325–330.

Vescovi AL, Galli R, Reynolds BA. 2006. Brain tumour stem cells. Nat Rev Cancer 6(6):425–436.

Ward WW, Bokman SH. 1982. Reversible denaturation of aequorea green-fluorescent protein – Physical separation and characterization of the renatured protein. Biochemistry 21(19):4535–4540.

Yang F, Moss LG, Phillips GN. 1996. The molecular structure of green fluorescent protein. Nat Biotechnol 14(10):1246–1251.

Zeviani M, Di Donato S. 2004. Mitochondrial disorders. Brain 127:2153–2172.
