# Supplementary material for: Engineered Polymeric Materials for Biological Applications: Overcoming Challenges of the Bio–Nano Interface
Source: Polymers (Basel). 2019 Sep 2;11(9):1441. doi: 10.3390/polym11091441 (PMC6780590; doi:10.3390/polym11091441)
Supplement: Supplementary File 1 [file polymers-11-01441-s001.zip › Escape Figure Permission/Escape figure citation/Hu Irvina 2007 Nano Letters Paper.pdf]

NANO LETTERS

2007
Vol. 7, No. 10
3056−3064

Cytosolic Delivery of Membrane-Impermeable Molecules in Dendritic Cells Using pH-Responsive Core–Shell Nanoparticles

Yuhua Hu,[†] Tamara Litwin,[‡] Arpun R. Nagaraja,[§] Brandon Kwong,[||] Joshua Katz, Nicki Watson,[⊥] and Darrell J. Irvine*,[§,||]

Department of Chemical Engineering, Massachusetts Institute of Technology, 77 Massachusetts Avenue, Cambridge, Massachusetts 02139, Department of Chemistry, Massachusetts Institute of Technology, 77 Massachusetts Avenue, Cambridge, Massachusetts 02139, Department of Materials Science and Engineering, Massachusetts Institute of Technology, 77 Massachusetts Avenue, Cambridge, Massachusetts 02139, Department of Biological Engineering, Massachusetts Institute of Technology, 77 Massachusetts Avenue, Cambridge, Massachusetts 02139, and Whitehead Institute, 9 Cambridge Center, Cambridge, Massachusetts 02142

Received June 28, 2007; Revised Manuscript Received September 6, 2007

Downloaded via UNIV OF MELBOURNE on May 22, 2019 at 06:31:30 (UTC).
See https://pubs.acs.org/sharingguidelines for options on how to legitimately share published articles.

ABSTRACT

Polycations that absorb protons in response to the acidification of endosomes can theoretically disrupt these vesicles via the "proton sponge" effect. To exploit this mechanism, we created nanoparticles with a segregated core–shell structure for efficient, noncytotoxic intracellular drug delivery. Cross-linked polymer nanoparticles were synthesized with a pH-responsive core and hydrophilic charged shell designed to disrupt endosomes and mediate drug/cell binding, respectively. By sequestering the relatively hydrophobic pH-responsive core component within a more hydrophilic pH-insensitive shell, nontoxic delivery of small molecules and proteins to the cytosol was achieved in dendritic cells, a key cell type of interest in the context of vaccines and immunotherapy.

Many potentially powerful therapeutic strategies for the treatment of disease require the delivery of drugs into the cytosolic or nuclear compartments of cells. Examples include gene therapy mediated by plasmid DNA,[1−3] gene silencing or RNA interference via oligonucleotides,[4] antitumor toxin delivery,[5−7] and therapeutic protein delivery.[8,9] Each of these examples requires the delivery of membrane-impermeable molecules into the cytosol. Cells may take up macromolecular drugs via endocytosis, macropinocytosis, or phagocytosis, but these processes confine the internalized compounds to closed vesicles (endosomes or phagosomes), where the pH is progressively lowered to 5.5−6.5.[1] The fusion of these vesicles with lysosomes, intracellular compartments carrying the degradation machinery of the cell at a pH as low as 4.5,[10,11] often leads to rapid destruction of therapeutic molecules with little or no release into the cytosol. Cytosolic delivery of membrane-impermeable molecules into dendritic cells (DCs), immune cells critically involved in the initiation of adaptive immune responses,[12−15] is of particular interest. DCs bind peptides derived from pathogens to their major histocompatibility complex (MHC) molecules, and present these peptide−MHC complexes at their surface for recognition by naïve T cells. Importantly, presentation of antigens to cytotoxic T cells is greatly amplified (up to 1000-fold) by delivery of antigens to the cytosol, where the DC intracellular machinery can load them efficiently onto class I MHC molecules for presentation to $CD8^+$ T cells.[16,17] Likewise, certain immunostimulatory molecules, such as mimics of viral RNA that trigger potent antiviral immune responses, operate by binding to proteins in the cytosol of DCs.[18] Finally, efficient cytosolic drug delivery in DCs could be used to deliver plasmid DNA or gene silencing reagents in order to amplify or suppress adaptive immune responses

* Corresponding author. E-mail: djirvine@mit.edu. Telephone: 617-452-4174. Fax: 617-452-3293.
† Department of Chemical Engineering, Massachusetts Institute of Technology.
‡ Department of Chemistry, Massachusetts Institute of Technology.
§ Department of Materials Science and Engineering, Massachusetts Institute of Technology.
|| Department of Biological Engineering, Massachusetts Institute of Technology.
⊥ Whitehead Institute.

10.1021/nl071542i CCC: $37.00 © 2007 American Chemical Society
Published on Web 09/21/2007

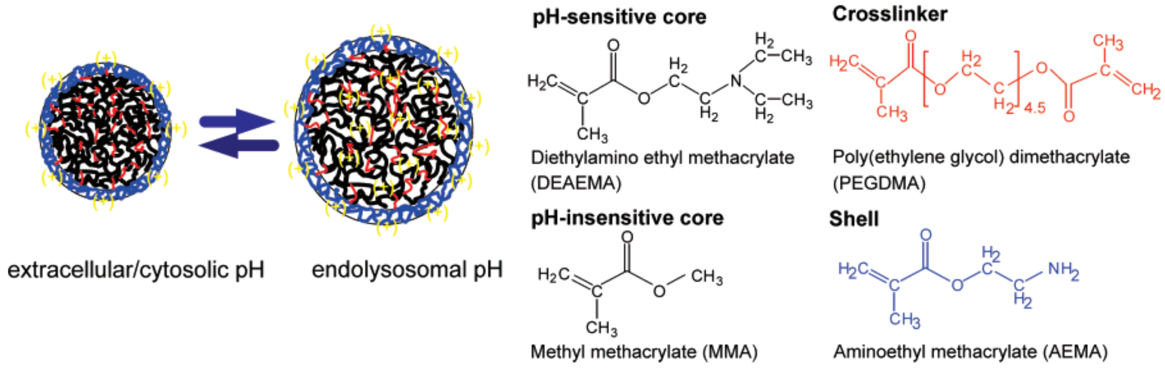

Figure 1. Schematic structure and chemical composition of pH-responsive core−shell nanoparticles. At extracellular/cytosolic pH, tertiary amines of DEAEMA repeat units in the particle cores are largely uncharged, and the particles are collapsed; at endolysosomal pH, the core tertiary amines ionize, and the particles swell. Surfactant-free polymerization of DEAEMA or MMA formed the core structure of hydrogel nanoparticles, crosslinked by PEGDMA; AEMA was polymerized in a second stage to form a thin shell structure rich in primary amines.

for vaccines or immunotherapy.[19] However, transfection of DCs is notoriously inefficient.[20−22]

To enable delivery of membrane-impermeable molecules into the cytosol of cells (as an endpoint destination or as a first step prior to trafficking to the nucleus), much research has been directed at the development of synthetic chaperones that can facilitate transport of hydrophilic molecules to the cytosol.[23] Approaches include the use of membrane-penetrating peptides,[24,25] pathogen-derived pore-forming proteins,[26,27] and "endosome escaping" polymers or lipids that disrupt the endosomal membrane in response to the pH reduction, which occurs in these compartments.[28−35] While many of these approaches show promise, strategies that can promote highly efficient delivery of molecules into the cytosol while avoiding unacceptable cytotoxicity are still sought. In addition, many of the chaperone molecules that efficiently aid transport of macromolecules into the cytosol are formulated with drug cargos by physical complexation of the chaperone and drug (e.g., polyplexes or lipoplexes of cationic polymers/lipids with DNA), forming nanoparticles whose size, stability, and properties are highly dependent on formulation parameters including the identity of the drug cargo, the drug-to-chaperone weight ratio, and the characteristics of the surrounding environment (pH, ionic strength, and presence/absence of serum proteins).[28,36] Lack of control over chaperone/drug particle size and stability is of concern because particle size is a critical determinant of cellular uptake in vitro and biodistribution and toxicity in vivo.[28]

We tested here an alternative strategy for cytosolic delivery, using monodisperse crosslinked hydrogel nanoparticles as chaperones for delivery of molecules to the cytosol. The chemical structure and proposed action of these nanoparticles is illustrated in Figure 1. We pursued a core−shell particle structure to physically and compositionally segregate the functions of the particle into an endosome-disrupting pH-responsive core that would absorb protons at endolysosomal pH and a shell whose composition could be separately tuned to facilitate particle targeting, cell binding, and/or drug binding. We utilized emulsion polymerization for the synthesis of these nanoparticles, which enables (1) a broad palette of chemical groups to be easily incorporated and (2) controlled fabrication of monodisperse nanoparticles of a predefined size. We found that these nanoparticles promoted highly efficient cytosolic delivery of both small molecules and macromolecules in dendritic cells in vitro.

The particles were synthesized by a two-stage surfactant-free emulsion polymerization in water. In the first stage, 2-diethylamino ethyl methacrylate (DEAEMA), which possesses a tertiary amine with a pK_b of 7.0−7.3,[37−40] was polymerized for 3 h with poly(ethylene glycol) dimethacrylate (PEGDMA, MW_{PEO} = 200 g/mol) as a crosslinker to form the pH-sensitive core of the particles (Figure 1; a detailed synthesis protocol is provided in the Supporting Information). Control particles were synthesized by replacing DEAEMA with the nonionizable monomer methyl methacrylate (MMA). In the second stage, 2-aminoethyl methacrylate (AEMA) was added to the stirring latex suspension to polymerize a pH-insensitive hydrophilic shell layer rich in primary amines (Figure 1). The polymerization was stopped after another 1.5 h, and the particles were purified by extensive dialysis and centrifugation/washing. To enable tracking of the nanoparticles by fluorescence microscopy, the fluorochrome cy5 succinimidyl ester was covalently conjugated to the primary amines available in the particle shells. As expected, the emulsion polymerization yielded highly monodisperse particles. The particles used in our cell studies were 205 ± 5 nm in diameter as observed in cryoEM (Figure 2A), which was in good agreement with the diameter of 208 ± 4 nm determined by dynamic light scattering (DLS, Brookhaven 90Plus instrument) at 37 °C in aqueous phosphate buffer at pH 7.4 and physiological ionic strength (Figure 2B).

The group of Armes has shown that PDEAEMA-containing latex particles exhibit sharp swelling transitions near neutral pH,[41,42] analogous to bulk hydrogels composed of weak polybase network chains.[43] To characterize the pH sensitivity of our core−shell nanoparticles, the hydrodynamic diameters (determined by DLS) were measured for particles equilibrated in physiological ionic strength phosphate buffers with pH ranging from 4.9 to 9.5 at 25 or 37 °C (Figure 2B). Particle swelling/deswelling equilibrated within 10 min and was reversible in response to changes in buffer pH. As shown in Figure 2B, PDEAEMA core−shell nanoparticles were largely deswollen at elevated pH but swelled abruptly

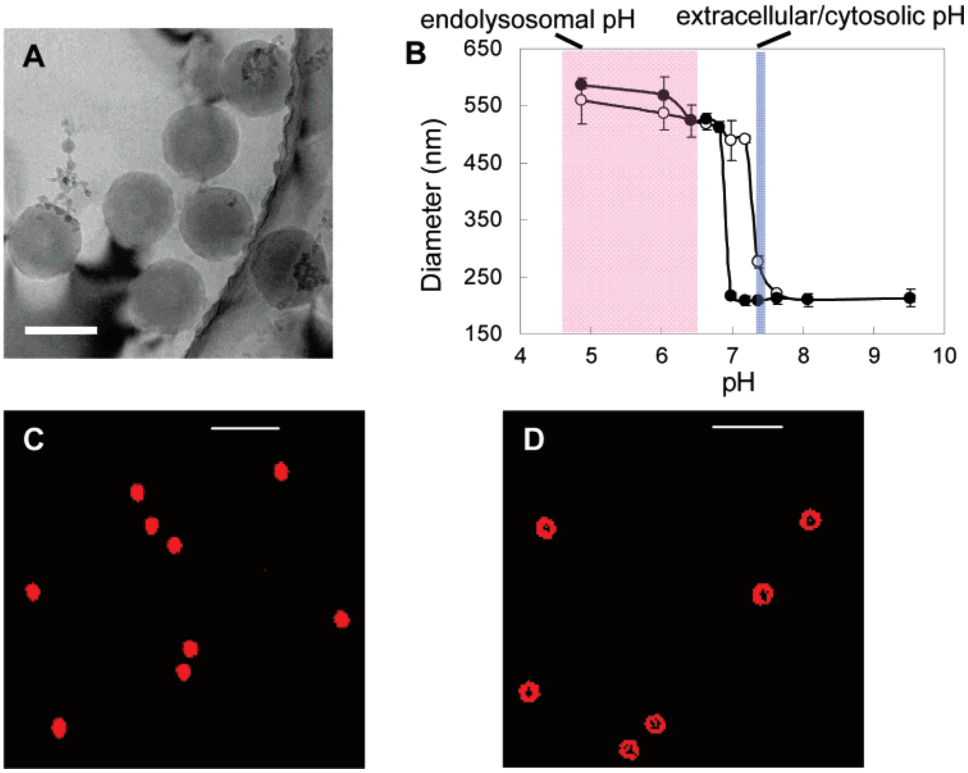

Figure 2. Morphology and pH-responsive swelling of core−shell nanoparticles. (A) CryoEM image of PDEAEMA-core/PAEMA-shell nanoparticles. Scale bar 200 nm. (B) Hydrodynamic diameters of the nanoparticles equilibrated in 100 mM phosphate buffers of different pHs, determined by DLS at (○) 25 or (●) 37 °C. (C, D) CLSM fluorescence images of PDEAEMA-core/PAEMA-shell nanoparticles with cy5 fluorophore conjugated to the shell of the particles at pH 7.5 (C) or pH 4.5 (D) in 100 mM phosphate buffer at 25 °C. Scale bars 5 μm.

between pH 7.0 and 6.8 at 37 °C. The particles exhibited a 2.8-fold change in diameter (by DLS) on moving from the extracellular/cytosolic pH of 7.4 to an endolysosomal pH of 5, corresponding to a ∼22-fold volume change. Similar swelling trends were observed by measuring the swelling ratio (hydrated mass/dry mass, data not shown). Note that the primary amines of the AEMA groups ($pK_b \sim 11$) in the particle shells should remain highly ionized across this entire pH range; only the tertiary amines of the particle cores will respond to the changes in pH. The swelling response of DEAEMA-containing nanoparticles showed modest temperature sensitivity: at 25 °C, the swelling transition was detected at pH ∼ 7.4, while at 37 °C, the swelling transition moved to pH ∼ 7.0. Control nanoparticles with pH-insensitive PMMA cores and PAEMA shells (diameter of 284 ± 11 nm as determined by DLS at pH 7.4) exhibited no size/swelling change in aqueous phosphate buffers having pH 4.5−9.5 (data not shown). The morphology of PDEAEMA-core nanoparticles with fluorophore-labeled shells was also directly observed at extracellular/cytosolic and endolysosomal pH using confocal laser scanning microscopy (CLSM). At pH 7.5 in the deswollen state (Figure 2C), the shell-labeled particles appeared as punctuate spheres, while particles incubated in pH 4.5 phosphate buffer swelled, with the fluorescent shell of the swollen nanoparticles clearly resolved (Figure 2D). The swelling measurements indicated a sharp onset of particle core ionization at endosomal pH, but the proton sponge mechanism of endosomal escape relies on the buffering capacity of polymers undergoing ionization.[44] Titration of initially basic aqueous particle suspensions with HCl revealed that the PDEAEMA-core particles had a substantial buffering capacity near neutral pH (Figure S1, Supporting Information). At 25 °C, the PDEAEMA cores of nanoparticles bound up to 0.46 mol H^+ per mole of DEAEMA units, buffering acidified solution near a pH ∼ 7.1.

As expected based on the incorporation of chemically stable PEGDMA cross-links, we found that the particles were stable at least one week in neutral saline; the particles swelled slightly over a week but retained a narrow size distribution (Figure S2, Supporting Information). The dry weight of the particles was essentially unchanged after one week in PBS (1.5 ± 0.7% weight loss, based on triplicate samples). In addition, the particles exhibited a nearly identical size distribution in pH 7.4 serum-free medium containing 10 mM glutathione (GSH) to mimic the reducing environment of the cytosol (Figure S2, Supporting Information).

We hypothesized that the pH sensitivity of these core−shell nanoparticles would facilitate endosome/phagosome disruption. Buffering polymers are thought to disrupt acidifying endosomes via an osmotic pressure buildup associated with chloride accumulation (the proton sponge effect).[45,46] In addition, membrane disruption might further be aided by swelling of the particles in situ. Because of our interest in delivering membrane-impermeable molecules into dendritic cells for vaccines and antiviral drug delivery, we investigated the uptake of the nanoparticles by a dendritic cell clone,[47] DC2.4. DCs are highly sensitive to the presence of lipopolysaccharide (LPS, also known as endotoxin), which can trigger activation of these cells through Toll-like receptor-

4. Nanoparticles were thus prepared using endotoxin-free water/buffers. We confirmed that the endotoxin contamination in these particles was below levels reported to stimulate DCs[48−50] (0.0027 EU/μg nanoparticles, or 0.0685 $\pm$ 0.001 EU/mL at the concentrations of nanoparticles used in our experiments; see Supporting Information).

Calcein, a membrane-impermeant fluorophore, was used as a model drug molecule and tracer to monitor the stability of endosomes/phagosomes following particle uptake.[31] DC2.4 cells (1.2 $\times$ 10^5 cells/well) were plated in Lab-Tek chambers for 18 h, and then calcein was added to the cells (150 μg/mL, 0.24 mM) with or without 25 μg/mL of PDEAEMA-core/PAEMA-shell or PMMA-core/PAEMA-shell nanoparticles in complete medium (RPMI 1640 with 10% fetal bovine serum (FBS)) for 1 h at 37 °C. After three washes with medium to remove extracellular calcein/particles, the cells were imaged live by CLSM at 37 °C. As shown in Figure 3A,D,G, cells treated with calcein alone showed a punctuate distribution of fluorescence indicative of endolysosomal compartmentalization of the dye. In contrast, cells co-incubated with calcein and PDEAEMA-core/PAEMA-shell nanoparticles exhibited calcein fluorescence throughout the cytosol and nucleus (Figure 3B,E,H). Calcein entry into the cytosol triggered by the presence of nanoparticles required the pH-sensitive core, as calcein remained in an endosomal distribution in cells co-incubated with calcein and PMMA-core/PAEMA-shell nanoparticles (Figures 3C,F,I). As shown in the confocal images (Figure 3E,F,H,I), both PDEAEMA and PMMA core−shell particles were taken up by the phagocytic DC2.4 cells, and treatment of cells with 0.04 wt %/vol trypan blue to quench extracellular fluorescence[47,51] following particle incubation confirmed that particles associated with cells were in fact internalized (data not shown). Optical sectioning of cells treated with PDEAEMA-core/PAEMA-shell nanoparticles also provided further evidence that particles in cells exhibiting cytosolic calcein fluorescence were localized within cells rather than simply bound to the plasma membrane (Figure S3, Supporting Information). Particles prepared with PDEAEMA cores but lacking the PAEMA shell also triggered cytosolic entry of calcein (Figure S4, Supporting Information), suggesting that the non-pH-responsive cationic amine groups in the shells are not required for the calcein distribution seen in Figure 3E,H. Figure 3J summarizes the frequency of cells observed by CLSM exhibiting endosomal vs cytosolic/nuclear calcein distributions after 1 h incubation under the three experimental conditions. Fewer than 5% of cells incubated with calcein alone or calcein together with PMMA core−shell nanoparticles exhibited a cytosolic/nuclear calcein distribution, while ~90% of cells incubated with PDEAEMA core−shell particles had calcein distributed throughout the cytosol. Experiments performed with different incubation times showed that the cytosolic delivery of calcein occurred within 45 min and was observed for more than 95% of cells incubated with calcein and PDEAEMA core−shell nanoparticles by 90 min. DCs treated with nanoparticles for 1 h, washed, and then cultured for an additional 24 h showed that most or all of the nanoparticles remain within the cells for at least 1 day (Figure S5, Supporting Information). The cytosolic delivery of calcein triggered by core−shell nanoparticles was not limited to dendritic cells, as we observed similar results in murine embryonic fibroblasts (Figure S6, Supporting Information).

The intracellular distribution of calcein observed in the presence of PDEAEMA-core/PAEMA-shell nanoparticles could arise either due to the particles causing disruption of endosomes that contain calcein or via nanoparticles permeablizing the cell surface plasma membrane (note that calcein that reaches the cytosol is also able to freely enter the nucleus by diffusion[52]). Although proton-absorbing polymers and lipids have been proposed to cause escape of molecules into the cytosol following endocytosis and endosome acidification via the proton sponge effect,[45] it has also been shown that polycations such as polyethylenimine (PEI) and poly-L-lysine (PLL) have the ability to directly interact with the plasma membrane (the exterior cell surface), generating nanoscale transient pores that allow leakage of molecules into and out of the cytoplasm.[53] To examine the mechanism of cytosolic delivery of calcein by the core−shell nanoparticles, we tested calcein uptake by DC2.4 cells under additional conditions. First, to confirm that endocytosis of the nanoparticles/calcein was required for calcein delivery to the cytosol, DCs were equilibrated at 4 °C for 30 min to block endocytosis and then calcein alone or calcein and nanoparticles were added to the cells (same calcein and nanoparticle concentrations as described in Figure 3). Neither calcein nor nanoparticles were internalized by cells following incubations up to 3 h at 4 °C, although some particles bound to the plasma membrane of cells (Figure S7, Supporting Information), suggesting that calcein/nanoparticle uptake and calcein entry into the cytosol of DCs required the active process of endocytosis. Second, to test whether acidification of endolysomes is necessary for the pH-sensitive nanoparticles to facilitate calcein delivery to the cytosol, we incubated DCs with the H^+-ATPase inhibitor bafilomycin A1 (80nM)[45,54] for an hour at 37 °C before adding calcein and nanoparticles. Consistent with the proton sponge mechanism, bafilomycin inhibition of endosome acidification blocked nanoparticle-mediated calcein delivery to the cytosol (data not shown). Next, as a test of plasma membrane integrity during the incubation of DCs with core−shell nanoparticles at 37 °C,[55] we loaded DCs with the fluorescent dye fura-2AM (Invitrogen) according to the manufacturer's instructions. Fura-2AM enters cells as a membrane-permeable acetomethoxy ester but is cleaved by intracellular esterases following loading into the cell to form a membrane-impermeable product that is trapped in the cytosol. We incubated fura-loaded DCs with core−shell nanoparticles and imaged the fluorescence from fura over 3 h at 37 °C by videomicroscopy in 1 min intervals to determine if fura escaped to the surrounding medium. Fura fluorescence photobleached uniformly in cells over time, equally in the control fura-only and fura/nanoparticle co-incubation experiments; no sign of fura loss driven by the nanoparticles was detected (data not shown). On the basis of these experiments, we conclude that the nanoparticles deliver calcein to the cytosol of cells by co-endocytosis of

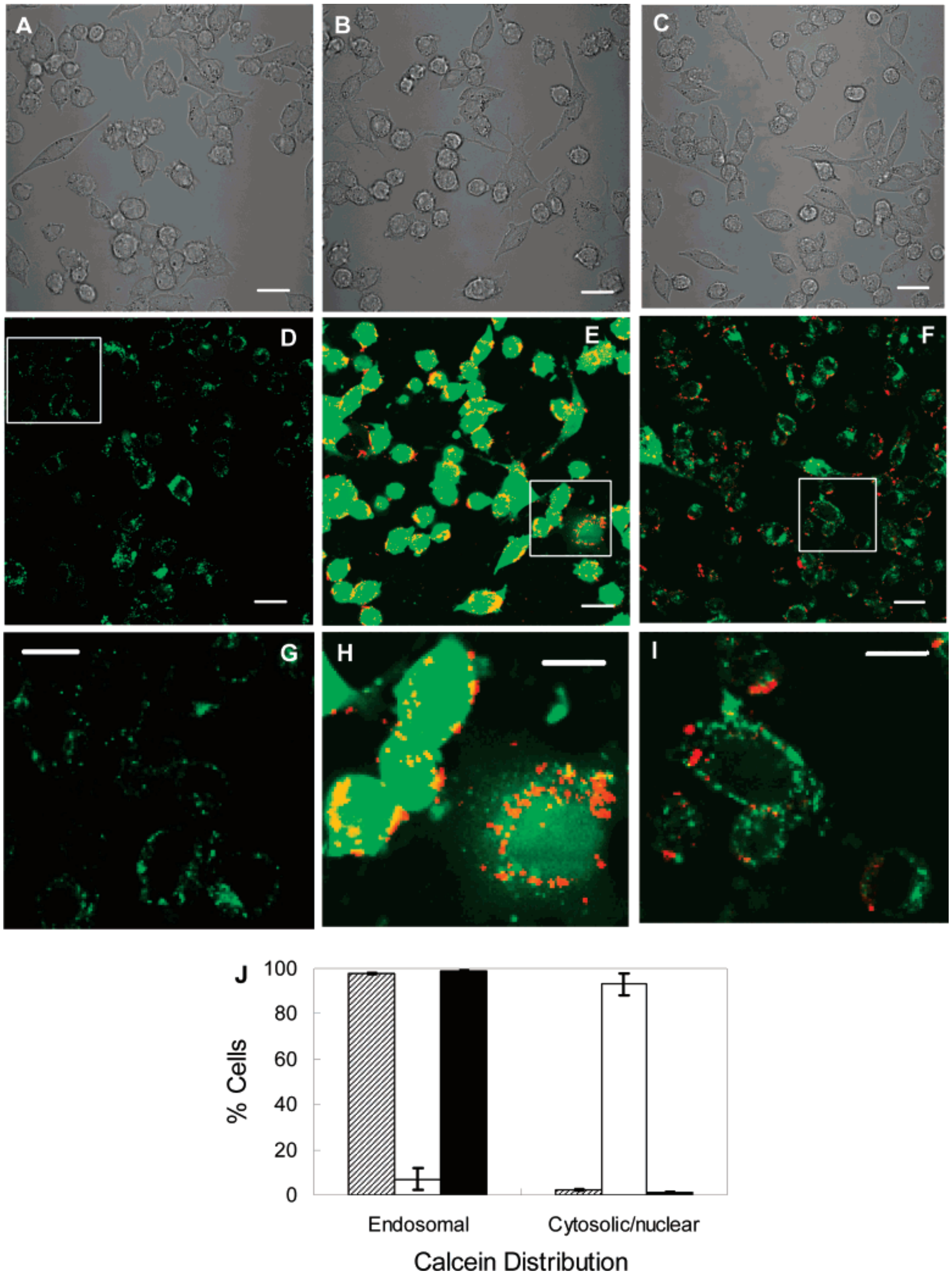

Figure 3. pH-responsive core−shell nanoparticles chaperone the delivery of the membrane-impermeable dye molecule calcein into the cytosol of dendritic cells. (A−I) CLSM images at 40×. (A−C) bright-field images. (D−I) Fluorescence overlays (red, nanoparticles; green, calcein). (A,D,G) Cells were treated with calcein alone. (B,E,H) Cells were co-incubated with calcein and PDEAEMA-core/PAEMA-shell nanoparticles. (C,F,I) Cells were co-incubated with calcein and PMMA-core/PAEMA-shell nanoparticles. Scale bars: (A−F) 20 μm; (G−I) 10 μm. (J) Average percentage of cells observed by CLSM exhibiting endosomal vs cytosolic/nuclear calcein distributions after 1 h from three independent experiments: calcein alone (gray bar), calcein with PDEAEMA core−shell particles (white bar), or calcein with PMMA core−shell particles (black bar) (n = 350−700 cells scored per condition in each experiment, *shown are means* ±SD).

calcein and particles, followed by particle disruption of endosomes and escape of the dye into the cytosol/nucleus.

To obtain more direct evidence for escape of the core−shell nanoparticles from endosomes into the cytosol, we performed confocal fluorescence imaging of cells incubated with nanoparticles in the presence of a fluorescent marker of endolysosomal compartments as well as transmission electron microscopy (TEM) on fixed thin sections of cells incubated with nanoparticles. The pH-sensitive fluorescent indicator LysoTracker Red DND-99 (1 μM, Invitrogen) was added together with 0.24 mM calcein and 25 μg/mL of core−shell nanoparticles to DC2.4 cells for 1 h at 37 °C in order to label endolysosomal compartments during calcein uptake. After washing, cells were imaged by CLSM at 37 °C. The CLSM images (Figure 4A,B,C) revealed that a significant fraction of the internalized PDEAEMA-core/PAEMA-shell nanoparticles (blue) failed to co-localize with endolysosomal vesicles (red) while delivering calcein (green) to the cytosol.

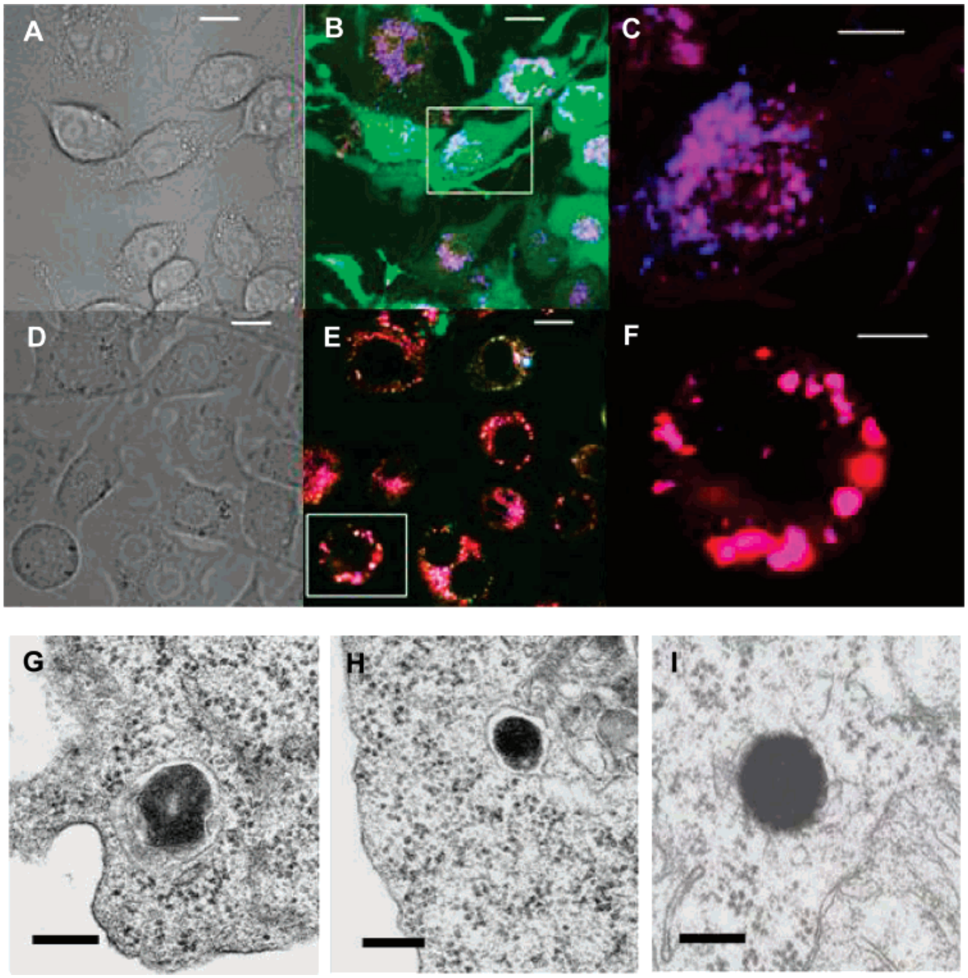

Figure 4. Endosomal escape of pH-responsive core−shell nanoparticles. (A−F) DC2.4 cells were co-incubated with LysoTracker Red DND-99 (to label endolysosomes), calcein, and either PDEAEMA-core/PAEMA-shell (A−C) or PMMA-core/PAEMA-shell nanoparticles (D−F). Confocal images were taken at 100× collecting bright-field images (A,D) as well as fluorescence (B,C,E,F) from calcein (green), LysoTracker Red (red), and cy5-labeled nanoparticles (blue). Scale bars 10 μm (A,B,D,E) and 5 μm (C,F, zoomed views of boxed areas in B,E showing overlaid lysotracker and nanoparticles fluorescence). (G−I) TEM images of nanoparticle localization within DC2.4 cells: (G) Cell sections with PMMA-core/PAEMA-shell particles revealed the particles internalized in membrane-bound compartments. (H−I) Cell sections with PDEAEMA-core/PAEMA-shell particles showed particles internalized either in membrane-bound compartments (H) or in the cell cytosol without a clear binding membrane structure (I). Scale bars 500 nm.

In contrast, cells co-incubated with calcein and PMMA-core/PAEMA-shell nanoparticles exhibited strong colocalization of nanoparticles (blue), endolysosomal vesicles (red), and calcein (green), which had a punctuate vesicular distribution (Figure 4D,E,F). To more directly interrogate the location of internalized nanoparticles, TEM images were taken of thin sections (50 nm) from fixed and stained cells following 1 h incubation of DC2.4 cells with nanoparticles (detailed sample preparation described in the Supporting Information). Core−shell nanoparticles with a pH-insensitive PMMA core were localized within membrane-bounded endosomes/phagosomes (Figure 4G). However, PDEAEMA core−shell nanoparticles were observed both within membrane-bounded vesicles (Figure 4H) and within the cytosol (Figure 4I). These trends were consistently observed in imaging 55 particles from three individual experiments (8 PMMA particles, and 47 PDEAEMA particles in which 10 were in endosomes and 37 were in the cytosol). Together, these data support the conclusion that the pH-sensitive PDEAEMA core−shell nanoparticles do not remain trapped in acidic intracellular compartments but instead escape to the cytosol following internalization.

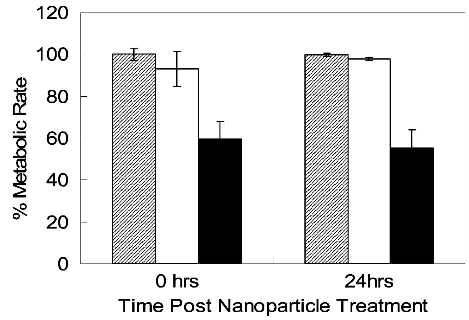

Figure 5. Metabolic rate of nanoparticle-treated cells relative to untreated controls. DC2.4 were incubated with PMMA-core/PAEMA-shell nanoparticles (gray bar), PDEAEMA-core/PAEMA-shell particles (white bar), or PDEAEMA-core/no-shell nanoparticles (black bar) for 1 h at 37 °C, washed, and then acute (0 h) and latent (24 h) effects on cell metabolism were measured by MTT assay. Error bars represent standard deviation of triplicate samples.

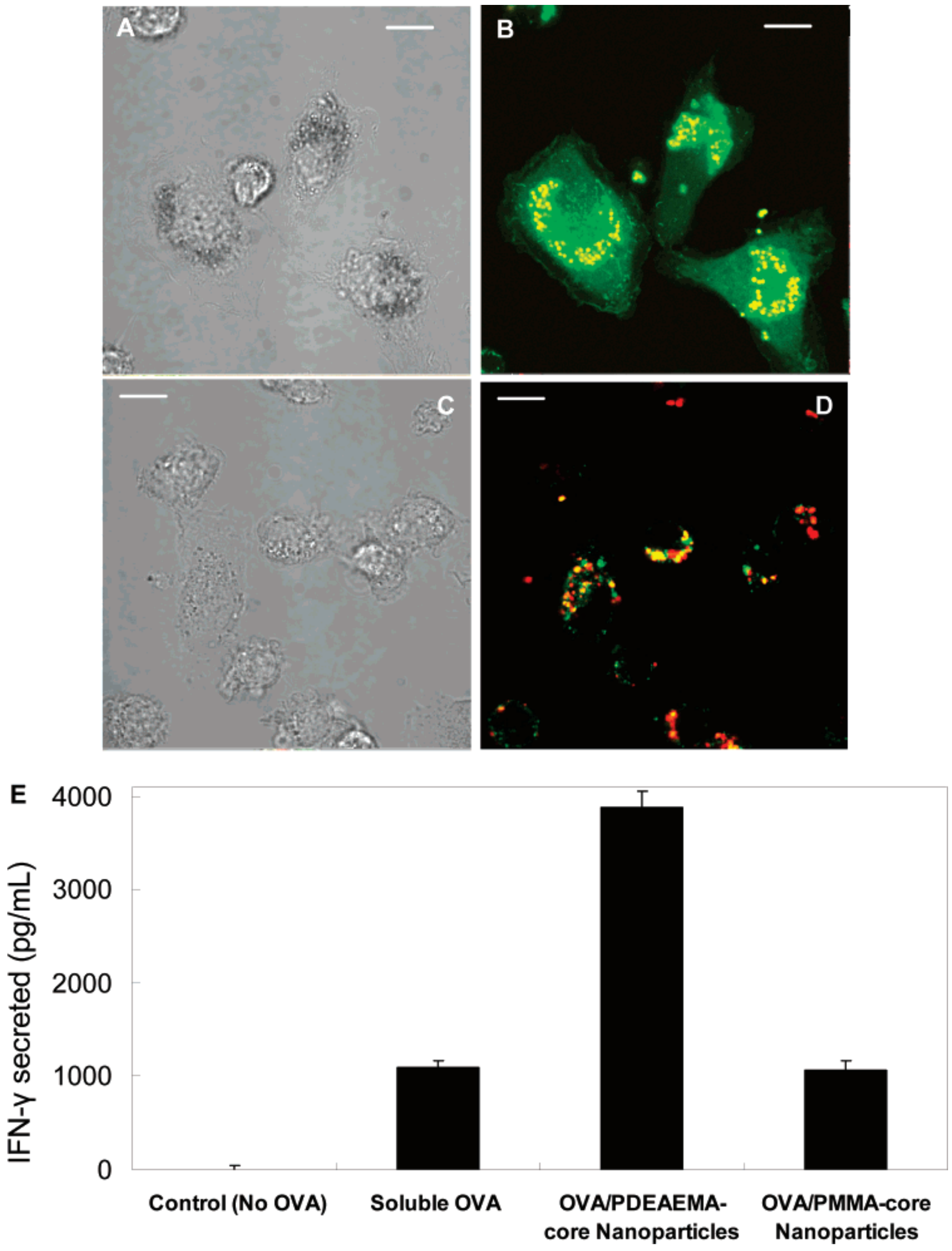

Figure 6. pH-sensitive core−shell nanoparticles deliver OVA to the cytosol of primary dendritic cells and promote $CD8^+$ T cell priming. (A−D) CLSM images at 100×: (A,C) Bright-field images; (B,D) Fluorescence overlays of OVA (green) and nanoparticles (red). (A,B) BMDCs incubated with OVA adsorbed to PDEAEMA core−shell nanoparticles. (C,D) Cells incubated with OVA adsorbed to PMMA core−shell nanoparticles. Scale bars 10 μm. (E) BMDCs were incubated with medium alone (no OVA), soluble OVA, OVA-coated PDEAEMA-core nanoparticles, or OVA-coated PMMA-core nanoparticles, then washed and mixed with naïve OT-1 OVA-specific $CD8^+$ T cells. IFN-γ secreted by the T cells in response to antigen presentation by the DCs was measured by ELISA after 72 h. Error bars represent standard deviation of triplicate samples.

A concern for the design of any intracellular delivery system is the cytotoxicity of the delivery material. To assess the cytotoxicity of the core−shell nanoparticles, a dimethylthiazolyl diphenyltetrazolium bromide (MTT) assay was used to determine the metabolic rate of cells exposed to nanoparticles vs untreated controls. Here, 5×10^4 DC2.4 cells were plated in triplicate in 96-well plates and incubated with or without 25 μg/mL of nanoparticles for 1 h at 37 °C. The cells were then washed with warm complete medium 3×. The metabolic rate (expressed as a percentage relative to controls that were not exposed to nanoparticles) was measured either immediately by the standard MTT assay (details in the Supporting Information) to detect the acute effects of nanoparticles on the cells or after an additional 24 h of culture to measure the latent effects of nanoparticle treatment on cell metabolism (Figure 5). Core−shell particles with a pH-insensitive PMMA core had negligible cytotoxicity up to 24 h post particle incubation. Likewise, core−shell nanoparticles with the pH-responsive PDEAEMA core also had very low toxicity: at nanoparticle concentrations of 25 μg/mL that provided efficient intracellular delivery of calcein shown in Figures 3−4, cells had ~95% of the metabolic

activity of controls at either tested time point. Notably, nanoparticles lacking a PAEMA-rich shell (PDEAEMA core only) exhibited much higher cytotoxicity than core−shell particles. This may reflect the combined impact of cationic charge and hydrophobicity in PDEAEMA, which is exposed in the "core-only" particles but sequestered in the core−shell structures under the more hydrophilic PAEMA surface layer; the combination of cationic charge and hydrophobicity is a common feature of highly membrane-interactive polymers.[56,57] Similar viability trends were obtained from a colony-forming assay, in which the ability of cells to grow for 3 days was measured after treatment with or without nanoparticles and replating into fresh culture wells (Figure S8, Supporting Information).

Having observed efficient cytosolic delivery of calcein and low cytotoxicity of DCs following treatment with the pH-sensitive core−shell nanoparticles, we finally tested the ability of these particles to chaperone the cytosolic delivery of a protein and promote a functional response. For these experiments, we employed bone marrow-derived dendritic cells (BMDCs) to determine whether delivery in primary cells differed from results obtained using the DC2.4 cell line. We first tested delivery of the model protein antigen ovalbumin (OVA, 45 KDa). OVA is known to be internalized by cells into endosomal compartments and has also been commonly used as an endocytic tracer.[58,59] Fluorescent OVA (100 μg/mL) was premixed with core−shell nanoparticles (25 μg/mL) for 5 min to allow electrostatic adsorption of the protein to the cationic surfaces of the core−shell particles. The majority of free OVA was removed by centrifugation and aspiration of supernatant. The OVA-adsorbed particles were then resuspended in complete medium and added to BMDCs for 1 h at 37 °C, followed by washing and confocal imaging. Similar to the prior results obtained for calcein, OVA fluorescence was observed throughout the cytosol and nucleus in BMDCs co-incubated with OVA-coated PDEAEMA core−shell nanoparticles (Figure 6A,B). The frequency of cells with cytosolic OVA was ∼43% ($n = 150$). When BMDCs were incubated with OVA-coated PMMA core−shell nanoparticles, the fluorescence was instead observed with a punctuated distribution in the endosomal compartments (Figure 6C,D). Similar results were obtained with DC2.4 cells (Figure S9, Supporting Information). Notably, because OVA was tightly absorbed on the surface of the core−shell nanoparticles due to the positively charged amine groups in the PAEMA shell, a mechanism for releasing OVA from the nanoparticles may be necessary to further enhance the frequency of cells exhibiting cytosolically distributed protein; studies addressing this issue are ongoing.

DCs can internalize protein antigens by endocytosis and break them down into peptides. When incubated with high concentrations of soluble antigens, dendritic cells have the ability to load peptides derived from a small fraction of such exogenously derived antigen onto class I MHC molecules, a process known as cross presentation. DCs displaying antigen bound to MHC I molecules on their surfaces can then activate $CD8^+$ T cells. However, direct delivery of protein antigens to the cytosol of DCs can substantially enhance the presentation of antigen on MHC I molecules. To determine whether nanoparticle-mediated transport of OVA to the cytosol could enhance priming of $CD8^+$ T cells by DCs, we treated BMDCs with 20 μg of soluble OVA or the same quantity of protein adsorbed to either PDEAEMA core−shell nanoparticles or PMMA core−shell nanoparticles for 1 h. Antigen/nanoparticle-loaded DCs were then mixed with naïve OT-1 $CD8^+$ T cells that specifically respond to a peptide derived from OVA[60] (details in the Supporting Information). Interferon-γ (IFN-γ) secreted by the T cells in response to antigen presentation by the DCs was measured by ELISA after 72 h. As shown in Figure 6E, some IFN-γ secretion was triggered by DCs incubated with soluble OVA, consistent with prior data from our laboratory and others showing that a small amount of spontaneous cross presentation can occur when DCs are incubated with this high concentration of soluble antigen.[51] However, DCs loaded with OVA via pH-responsive nanoparticles elicited 4-fold more IFN-γ from T cells. In contrast, DCs pulsed with OVA via pH-nonresponsive nanoparticles elicited the same level of IFN-γ as the soluble OVA control.

In conclusion, we synthesized pH-sensitive PDEAEMA-core/PAEMA-shell nanoparticles and demonstrated that these particles are capable of efficient cytosolic delivery of membrane-impermeable molecules such as calcein and OVA protein to dendritic cells. By sequestering the hydrophobic, pH-buffering component of the polymer particles within the core under a more hydrophilic shell composition, these particles effectively disrupted endosomes and delivered molecules to the cytosol of cells without overt cytotoxicity. These materials may be of utility for delivery of membrane impermeable drug compounds or oligonucleotides to the cytosol of dendritic cells for immunotherapy, and other cell types for cytosolic drug therapy. Presently, we are exploring the extension of this concept to core−shell nanoparticles that quickly dissolve to nontoxic soluble components upon reaching the cytosol to promote efficient unpacking of drugs carried to the cytosolic compartments.

Acknowledgment. This work was supported by the U.S. Army Research Office through the Institute for Soldier Nanotechnology, the Human Frontiers Science Program, and the National Science Foundation (award 0348259). We acknowledge Kazuyoshi Murata from the Whitehead-MIT BioImaging Center for his expert assistance on the CryoEM.

Supporting Information Available: Description of the materials, experimental details, and supplementary figures. This material is available free of charge via the Internet at http://pubs.acs.org.

References

(1) Medina-Kauwe, L. K.; Xie, J.; Hamm-Alvarez, S. *Gene Ther.* **2005**, *12*, 1734−1751.
(2) Putnam, D. *Nat. Mater.* **2006**, *5*, 439−451.
(3) Wagner, E.; Kloeckner, J. *Polym. Ther., Part I* **2006**, *192*, 135−173.
(4) Tagami, T.; Barichello, J. M.; Kikuchi, H.; Ishida, T.; Kiwada, H. *Int. J. Pharm.* **2007**, *333*, 62−69.
(5) Devalapally, H.; Shenoy, D.; Little, S.; Langer, R.; Amiji, M. *Cancer Chemother. Pharmacol.* **2007**, *59*, 477−484.

(6) Borghouts, C.; Kunz, C.; Groner, B. *J. Pept. Sci.* **2005**, *11*, 713−726.
(7) Son, Y. J.; Jang, J. S.; Cho, Y. W.; Chung, H.; Park, R. W.; Kwon, I. C.; Kim, I. S.; Park, J. Y.; Seo, S. B.; Park, C. R.; Jeong, S. Y. *J. Controlled Release* **2003**, *91*, 135−145.
(8) Schweichel, D.; Steitz, J.; Tormo, D.; Gaffal, E.; Ferrer, A.; Buchs, S.; Speuser, P.; Limmer, A.; Tuting, T. *J. Gene Med.* **2006**, *8*, 1243−1250.
(9) Determan, A. S.; Wilson, J. H.; Kipper, M. J.; Wannemuehler, M. J.; Narasimhan, B. *Biomaterials* **2006**, *27*, 3312−3320.
(10) Asokan, A.; Cho, M. J. *J. Pharm. Sci.* **2002**, *91*, 903−913.
(11) Akinc, A.; Langer, R. *Biotechnol. Bioeng.* **2002**, *78*, 503−508.
(12) Banchereau, J.; Steinman, R. M. *Nature* **1998**, *392*, 245−252.
(13) Banchereau, J.; Palucka, K.; Pulendran, B. *J. Invest. Dermatol.* **2000**, *114*, 207−207.
(14) Banchereau, J.; Briere, F.; Caux, C.; Davoust, J.; Lebecque, S.; Liu, Y. T.; Pulendran, B.; Palucka, K. *Annu. Rev. Immunol.* **2000**, *18*, 767.
(15) Palucka, K.; Banchereau, J. *J. Clin. Immunol.* **1999**, *19*, 12−25.
(16) Zarei, S.; Arrighi, J. F.; Ongaro, G.; Calzascia, T.; Haller, O.; Frossard, C.; Piguet, V.; Walker, P. K.; Hauser, C. *J. Invest. Dermatol.* **2003**, *121*, 745−750.
(17) Raychaudhuri, S.; Rock, K. L. *Nat. Biotechnol.* **1998**, *16*, 1025−1031.
(18) Diebold, S. S.; Montoya, M.; Unger, H.; Alexopoulou, L.; Roy, P.; Haswell, L. E.; Al-Shamkhani, A.; Flavell, R.; Borrow, P.; Sousa, C. R. E. *Nature* **2003**, *424*, 324−328.
(19) Greenland, J. R.; Geiben, R.; Ghosh, S.; Pastor, W. A.; Letvin, N. L. *J. Immunol.* **2007**, *178*, 5652−5658.
(20) Awasthi, S.; Cox, R. A. *Biotechniques* **2003**, *35*, 600.
(21) Irvine, A. S.; Trinder, P. K. E.; Laughton, D. L.; Ketteringham, H.; McDermott, R. H.; Reid, S. C. H.; Haines, A. M. R.; Amir, A.; Husain, R.; Doshi, R.; Young, L. S.; Mountain, A. *Nat. Biotechnol.* **2000**, *18*, 1273−1278.
(22) Yoshinaga, T.; Yasuda, K.; Ogawa, Y.; Takakura, Y. *Biochem. Biophys. Res. Commun.* **2002**, *299*, 389−394.
(23) Uhrich, K. E.; Cannizzaro, S. M.; Langer, R. S.; Shakesheff, K. M. *Chem. Rev.* **1999**, *99*, 3181−3198.
(24) Rinne, J.; Albarran, B.; Jylhava, J.; Ihalainen, T. O.; Kankaanpaa, P.; Hytonen, V. P.; Stayton, P. S.; Kulomaa, M. S.; Vihinen-Ranta, M. *BMC Biotechnol.* **2007**, *7*, 1.
(25) Moelle, K.; Tada, Y.; Shibagaki, N.; Knop, J.; Udey, M. C.; von Stebut, E. *J. Invest. Dermatol.* **2006**, *126*, 130−130.
(26) Provoda, C. J.; Stier, E. M.; Lee, K. D. *J. Biol. Chem.* **2003**, *278*, 35102−35108.
(27) Cabiaux, V. *Adv. Drug Delivery Rev.* **2004**, *56*, 987−997.
(28) Wightman, L.; Kircheis, R.; Rossler, V.; Carotta, S.; Ruzicka, R.; Kursa, M.; Wagner, E. *J. Gene Med.* **2001**, *3*, 362−372.
(29) Park, T. G.; Jeong, J. H.; Kim, S. W. *Adv. Drug Delivery Rev.* **2006**, *58*, 467−486.
(30) Pun, S. H.; Davis, M. E. *Bioconjugate Chem.* **2002**, *13*, 630−639.
(31) Jones, R. A.; Cheung, C. Y.; Black, F. E.; Zia, J. K.; Stayton, P. S.; Hoffman, A. S.; Wilson, M. R. *Biochem. J.* **2003**, *372*, 65−75.
(32) Little, S. R.; Lynn, D. M.; Ge, Q.; Anderson, D. G.; Puram, S. V.; Chen, J. Z.; Eisen, H. N.; Langer, R. *Proc. Natl. Acad. Sci. U.S.A.* **2004**, *101*, 9534−9539.
(33) Remaut, K.; Lucas, B.; Raemdonck, K.; Braeckmans, K.; Demeester, J.; De Smedt, S. C. *Biomacromolecules* **2007**, *8*, 1333−1340.
(34) Wasungu, L.; Hoekstra, D. *J. Controlled Release* **2006**, *116*, 255−264.
(35) Ewert, K. K.; Ahmad, A.; Evans, H. M.; Safinya, C. R. *Expert Opin. Biol. Ther.* **2005**, *5*, 33−53.
(36) Pack, D. W.; Hoffman, A. S.; Pun, S.; Stayton, P. S. *Nat. Rev. Drug Discovery* **2005**, *4*, 581−593.
(37) Podual, K.; Doyle, F. J.; Peppas, N. A. *Polymer* **2000**, *41*, 3975−3983.
(38) Podual, K.; Doyle, F. J.; Peppas, N. A. *Biomaterials* **2000**, *21*, 1439−1450.
(39) Podual, K.; Peppas, N. A. *Polym. Int.* **2005**, *54*, 581−593.
(40) Lee, A. S.; Gast, A. P.; Butun, V.; Armes, S. P. *Macromolecules* **1999**, *32*, 4302−4310.
(41) Amalvy, J. I.; Unali, G. F.; Li, Y.; Granger-Bevan, S.; Armes, S. P.; Binks, B. P.; Rodrigues, J. A.; Whitby, C. P. *Langmuir* **2004**, *20*, 4345−4354.
(42) Amalvy, J. I.; Wanless, E. J.; Li, Y.; Michailidou, V.; Armes, S. P.; Duccini, Y. *Langmuir* **2004**, *20*, 8992−8999.
(43) Plunkett, K. N.; Moore, J. S. *Langmuir* **2004**, *20*, 6535−6537.
(44) Funhoff, A. M.; van Nostrum, C. F.; Koning, G. A.; Schuurmans-Nieuwenbroek, N. M. E.; Crommelin, D. J. A.; Hennink, W. E. *Biomacromolecules* **2004**, *5*, 32−39.
(45) Sonawane, N. D.; Szoka, F. C.; Verkman, A. S. *J. Biol. Chem.* **2003**, *278*, 44826−44831.
(46) Zuidam, N. J.; Posthuma, G.; de Vries, E. T. J.; Crommelin, D. J. A.; Hennink, W. E.; Storm, G. *J. Drug Targeting* **2000**, *8*, 5.
(47) Shen, Z. H.; Reznikoff, G.; Dranoff, G.; Rock, K. L. *J. Immunol.* **1997**, *158*, 2723−2730.
(48) Jotwani, R.; Pulendran, B.; Agrawal, S.; Cutler, C. W. *Eur. J. Immunol.* **2003**, *33*, 2980−2986.
(49) Thiele, L.; Rothen-Rutishauser, B.; Jilek, S.; Wunderli-Allenspach, H.; Merkle, H. P.; Walter, E. *J. Controlled Release* **2001**, *76*, 59−71.
(50) Vallhov, H.; Qin, J.; Johansson, S. M.; Ahlborg, N.; Muhammed, M. A.; Scheynius, A.; Gabrielsson, S. *Nano Lett.* **2006**, *6*, 1682−1686.
(51) Jain, S.; Yap, W. T.; Irvine, D. J. *Biomacromolecules* **2005**, *6*, 2590−2600.
(52) Heinzen, R. A.; Hackstadt, T. *Infect. Immun.* **1997**, *65*, 1088−1094.
(53) Hong, S. P.; Leroueil, P. R.; Janus, E. K.; Peters, J. L.; Kober, M. M.; Islam, M. T.; Orr, B. G.; Baker, J. R.; Holl, M. M. B. *Bioconjugate Chem.* **2006**, *17*, 728−734.
(54) Yoshimori, T.; Yamamoto, A.; Moriyama, Y.; Futai, M.; Tashiro, Y. *J. Biol. Chem.* **1991**, *266*, 17707−17712.
(55) Drin, G.; Cottin, S.; Blanc, E.; Rees, A. R.; Temsamani, J. *J. Biol. Chem.* **2003**, *278*, 31192−31201.
(56) Rungsardthong, U.; Deshpande, M.; Bailey, L.; Vamvakaki, M.; Armes, S. P.; Garnett, M. C.; Stolnik, S. *J. Controlled Release* **2001**, *73*, 359−380.
(57) Rungsardthong, U.; Ehtezazi, T.; Bailey, L.; Armes, S. P.; Garnett, M. C.; Stolnik, S. *Biomacromolecules* **2003**, *4*, 683−690.
(58) Swanson, M. S.; Isberg, R. R. *Infect. Immun.* **1996**, *64*, 2585−2594.
(59) Refolo, L. M.; Sambamurti, K.; Efthimiopoulos, S.; Pappolla, M. A.; Robakis, N. K. *J. Neurosci. Res.* **1995**, *40*, 694−706.
(60) Clarke, S. R. M.; Barnden, M.; Kurts, C.; Carbone, F. R.; Miller, J. F.; Heath, W. R. *Immunol. Cell Biol.* **2000**, *78*, 110−117.

NL071542I
