# Supplementary material for: Engineered Polymeric Materials for Biological Applications: Overcoming Challenges of the Bio–Nano Interface
Source: Polymers (Basel). 2019 Sep 2;11(9):1441. doi: 10.3390/polym11091441 (PMC6780590; doi:10.3390/polym11091441)
Supplement: Supplementary File 1 [file polymers-11-01441-s001.zip › Escape Figure Permission/Fig 4. Permission - Hu, Irvine 2007 Rightslink⌐ by Copyright Clearance Center.pdf]

ACS Publications
Most Trusted. Most Cited. Most Read.

Title: Cytosolic Delivery of Membrane-Impermeable Molecules in Dendritic Cells Using pH-Responsive Core−Shell Nanoparticles

Author: Yuhua Hu, Tamara Litwin, Arpun R. Nagaraja, et al

Publication: Nano Letters

Publisher: American Chemical Society

Date: Oct 1, 2007

Copyright © 2007, American Chemical Society

PERMISSION/LICENSE IS GRANTED FOR YOUR ORDER AT NO CHARGE

This type of permission/license, instead of the standard Terms & Conditions, is sent to you because no fee is being charged for your order. Please note the following:

- Permission is granted for your request in both print and electronic formats, and translations.
- If figures and/or tables were requested, they may be adapted or used in part.
- Please print this page for your records and send a copy of it to your publisher/graduate school.
- Appropriate credit for the requested material should be given as follows: "Reprinted (adapted) with permission from (COMPLETE REFERENCE CITATION). Copyright (YEAR) American Chemical Society." Insert appropriate information in place of the capitalized words.
- One-time permission is granted only for the use specified in your request. No additional uses are granted (such as derivative works or other editions). For any other uses, please submit a new request.

If credit is given to another source for the material you requested, permission must be obtained from that source.

BACK | CLOSE WINDOW

Copyright © 2019 Copyright Clearance Center, Inc. All Rights Reserved. Privacy statement. Terms and Conditions.
Comments? We would like to hear from you. E-mail us at customercare@copyright.com
